# Measuring Dispersion and Serial Dependence in Ordinal Time Series Based on the Cumulative Paired *ϕ*-Entropy

**DOI:** 10.3390/e24010042

**Published:** 2021-12-26

**Authors:** Christian H. Weiß

**Affiliations:** Department of Mathematics and Statistics, Helmut Schmidt University, 22043 Hamburg, Germany; weissc@hsu-hh.de; Tel.: +49-40-6541-2779

**Keywords:** Cohen’s κ, dispersion, entropy, ordinal time series, serial dependence

## Abstract

The family of cumulative paired ϕ-entropies offers a wide variety of ordinal dispersion measures, covering many well-known dispersion measures as a special case. After a comprehensive analysis of this family of entropies, we consider the corresponding sample versions and derive their asymptotic distributions for stationary ordinal time series data. Based on an investigation of their asymptotic bias, we propose a family of signed serial dependence measures, which can be understood as weighted types of Cohen’s κ, with the weights being related to the actual choice of ϕ. Again, the asymptotic distribution of the corresponding sample $\kappa_{\phi }$ is derived and applied to test for serial dependence in ordinal time series. Using numerical computations and simulations, the practical relevance of the dispersion and dependence measures is investigated. We conclude with an environmental data example, where the novel ϕ-entropy-related measures are applied to an ordinal time series on the daily level of air quality.

## 1. Introduction

During the last years, ordinal data in general [1] and ordinal time series in particular [2] received a great amount of interest in research and applications. Here, a random variable *X* is said to be ordinal if *X* has a bounded qualitative range exhibiting a natural order among the categories. We denote the range as $S=\{s_{0},s_{1},\ldots ,s_{m}\}$ with some m∈N={1,2,…}, and we assume that $s_{0}<\ldots <s_{m}$. The realized data are denoted as $x_{1},\ldots ,x_{n}$ with n∈N. They are assumed to stem either from independent and identically distributed (i. i. d.) replications of *X* (then, we refer to $x_{1},\ldots ,x_{n}$ as an ordinal random sample), or from a stationary ordinal stochastic process ${\left(X_{t}\right)}_{Z=\{\ldots ,-1,0,1,\ldots \}}$ (then, $x_{1},\ldots ,x_{n}$ are said to be an ordinal time series).

In what follows, we take up several recent works on measures of dispersion and serial dependence in ordinal (time series) data. Regarding ordinal dispersion, the well-known measures such as variance or mean absolute deviation cannot be used as the data are not quantitative. Therefore, several tailor-made measures for ordinal dispersion have been developed and investigated in the literature, see, among others, [3,4,5,6,7,8,9,10,11,12,13,14,15]. The unique feature of all these measures is that they rely on the cumulative distribution function (CDF) of *X*, i.e., on $f={\left(f_{0},\ldots ,f_{m-1}\right)}^{⊤}$ with $f_{i}=P\left(X\leq s_{i}\right)$ for i=0,…,m ($f_{m}$ is omitted in ***f*** as it necessarily equals one). They classify any one-point distribution on S as a scenario of minimal dispersion, i.e., if all probability mass concentrates on one category from S (maximal consensus):

$$f_{one}\;\in \;\left\{\left(\begin{array}{c} 1\\ 1\\ \vdots \\ 1 \end{array}\right),\;\left(\begin{array}{c} 0\\ 1\\ \vdots \\ 1 \end{array}\right),\ldots ,\;\left(\begin{array}{c} 0\\ \vdots \\ 0\\ 1 \end{array}\right),\;\left(\begin{array}{c} 0\\ \vdots \\ 0\\ 0 \end{array}\right)\right\}.$$

By contrast, maximal dispersion is achieved exactly in the case of the extreme two-point distribution (polarized distribution), $f_{two}={\left(\frac{1}{2},\ldots ,\frac{1}{2}\right)}^{⊤}$, where we have 50 % probability mass in each of the outermost categories (maximal dissent). Further details on ordinal dispersion measures are presented in Section 2 below.

Building upon earlier works by Klein [16], Yager [17], it was recently shown by Klein Doll [18], Klein et al. [19] that the aforementioned ordinal dispersion measures can be subsumed under the family of so-called “cumulative paired ϕ-entropies” (see Section 2), abbreviated as ${\mathrm{CPE}}_{\phi }$, which constitutes the starting point of the present article. Our first main task is to derive the asymptotic distribution of the corresponding sample version, ${\hat{\mathrm{CPE}}}_{\phi }$, for both i. i. d. and time series data, and to check the finite sample performance of the resulting approximate distribution, see Section 3 and Section 5.

In the recent paper by Weiß [20] on the asymptotics of some well-known dispersion measures for nominal data (i.e., qualitative data without a natural ordering), it turned out that the corresponding dispersion measures—if these are applied to time series data—are related to specific measures of serial dependence. Therefore, our second main task is to explore for a similar relation in the ordinal case, if considering the ${\mathrm{CPE}}_{\phi }$-family for measuring dispersion. Ordinal dependence measures can be defined in analogy to the popular autocorrelation function (ACF) for quantitative time series, namely by using the lagged bivariate CDF $f_{ij}\left(h\right)=P\left(X_{t}\leq s_{i},X_{t-h}\leq s_{j}\right)$ for time lags h∈N as their base [14]. For the novel family of $\kappa_{\phi }\left(h\right)$ measures, which cover the existing ordinal Cohen’s κ [14,15] as a special case, we derive the asymptotics under the null hypothesis of i. i. d. time series data, see Section 4. This result is used in Section 5 to test for significant serial dependence, in analogy to the application of the sample ACF to quantitative time series. In Section 6, we discuss an illustrative real-world data example from an environmental application, namely regarding the daily level of air quality. The article concludes in Section 7.

## 2. The Family of Cumulative Paired ϕ-Entropies

Klein Doll [18], Klein et al. [19] proposed and investigated a family of cumulative paired ϕ-entropies. Although their main focus was on continuously distributed random variables, they also referred to the ordinal case and pointed out that many well-known ordinal dispersion measures are included in this family. Here, we exclusively concentrate on the ordinal case as introduced in Section 1, and we define the (normalized) cumulative paired ϕ-entropy as (see Section 2.3 in Klein et al. [19])

(1) $${\mathrm{CPE}}_{\phi }\left(f\right)\;=\;\frac{1}{2\;m\;\phi (1/2)}\;\sum_{i=0}^{m-1}\left(\phi \left(f_{i}\right)+\phi \left(1-f_{i}\right)\right).$$

The entropy generating function (EGF) ϕ is defined on [0;1], it satisfies ϕ(0)=ϕ(1)=0, and it is assumed to be concave on [0;1]. Later in Section 3, when deriving the asymptotic distribution of the sample counterpart ${\hat{\mathrm{CPE}}}_{\phi }={\mathrm{CPE}}_{\phi }\left(\hat{f}\right)$, we shall also require that ϕ is (twice) differentiable. As pointed out in Section 2.3 and 3.1 of Klein et al. [19], several well-known measures of ordinal dispersion can be expressed by (1) with an appropriate choice of ϕ.

- *Leik’s ordinal variation* [11] corresponds to the choice ϕ(z)=min{z,1−z} (which is not differentiable in z=1/2) because of the equality |2z−1|=1−2min{z,1−z}:
  (2) $$\mathrm{LOV}\;=\;1-\frac{1}{m}\;\sum_{i=0}^{m-1}\left|2\;f_{i}-1\right|\;=\;\frac{2}{m}\;\sum_{i=0}^{m-1}\min\left\{f_{i},1-f_{i}\right\}.$$
- An analogous argument applies to the whole family of ordinal variation measures, ${\mathrm{OV}}_{q}$ with q≥1 [3,9,10,13]. Choosing $\phi_{q}\left(z\right)=1-{\left(1-2\;\min\left\{z,1-z\right\}\right)}^{q}=1-{\left|2\;z-1\right|}^{q}$ with $\phi_{q}\left(1/2\right)=1$, we have the relation
  (3) $${\mathrm{OV}}_{q}\;=\;1-{\left(\frac{1}{m}\;\sum_{i=0}^{m-1}{\left|2f_{i}-1\right|}^{q}\right)}^{1/q}\;=\;1-{(1-{\mathrm{CPE}}_{\phi }\left(f\right))}^{1/q}.$$
  Note that q=1 leads to the LOV, and the case q=2 is known as the coefficient of ordinal variation, $\mathrm{COV}={\mathrm{OV}}_{2}$ [4,8].
- Related to the previous case ${\mathrm{OV}}_{q}$ with q=2, ${\mathrm{CPE}}_{\phi }\left(f\right)$ becomes the widely-used index of ordinal variation [3,7,8] if choosing ϕ(z)=z(1−z):
  (4) $$\mathrm{IOV}\;=\;\frac{4}{m}\;\sum_{i=0}^{m-1}f_{i}\left(1-f_{i}\right)\;=\;1-\frac{1}{m}\;\sum_{i=0}^{m-1}{\left(2f_{i}-1\right)}^{2}.$$
- The cumulative paired (Shannon) entropy [12] corresponds to the choice ϕ(z)=−zlnz (with the convention 0ln0=0):
  (5) $$\mathrm{CPE}\;=\;\frac{-1}{m\;\ln2}\;\sum_{i=0}^{m-1}(f_{i}\;\ln f_{i}+\left(1-f_{i}\right)\;\ln\left(1-f_{i}\right)).$$
- ϕ(z)=−zlnz can be embedded into the family of *a*-entropies [21,22],
  (6) $$\phi_{a}\left(z\right)\;=\;\frac{z-z^{a}}{a-1}\;\mathrm{with}\;a>0\;\mathrm{and}\;a\neq 1,$$
  as the boundary case a→1. Plugging-in (6) into (1), one obtains
  (7) $${\mathrm{CPE}}_{a}\;=\;\frac{2^{a-1}}{m\;(2^{a-1}-1)}\;\sum_{i=0}^{m-1}(1-f_{i}^{a}-{\left(1-f_{i}\right)}^{a}).$$
  Note that both a=2 and a=3 in (7) lead to the IOV (4).

**Figure 1:**
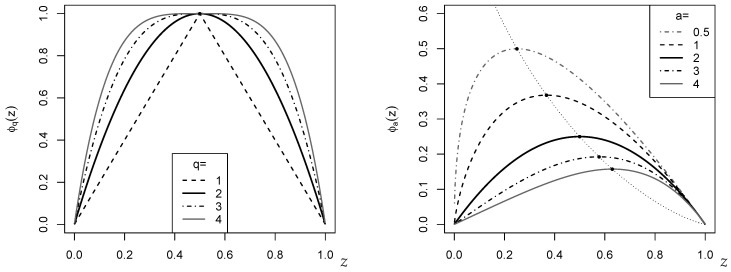
Plot of EGFs ϕ(z) against *z*. (**Left**): $\phi_{q}\left(z\right)=1-{\left|2\;z-1\right|}^{q}$; (**right**): $\phi_{a}\left(z\right)=\left(z-z^{a}\right)/\left(a-1\right)$.

The EGFs ϕ involved in (2)–(4) are symmetric around 1/2, i.e., they satisfy ϕ(z)=ϕ(1−z). This is also illustrated by Figure 1, where the cases q=2 (left) and a=2 (right; both in bold black) agree with each other except a scaling factor. The plotted EGFs $\phi_{q}\left(z\right)$ are maximal in 1/2 with $\phi_{q}\left(1/2\right)=1$. The EGF $\phi_{a}\left(z\right)$ is maximal in $a^{1/(1-a)}$ with $\phi_{a}\left(a^{1/(1-a)}\right)=a^{a/(1-a)}$ for a≠1, whereas in the boundary case a→1, $\phi_{a}\left(z\right)$ takes its maximal value 1/e at 1/e. However, since ${\mathrm{CPE}}_{\phi }$ in (1) is normalized, it is not necessary to care for a possible rescaling of $\phi_{a}\left(z\right)$ if computing ${\mathrm{CPE}}_{\phi }$.

**Remark** **1.** *The dotted curve in the right part of Figure 1, which connects the maxima of $\phi_{a}\left(z\right)$ for different a, is computed by using the Lambert W function (also referred to as the product logarithm). This can be seen as follows: $\phi_{a}\left(z\right)$ is maximal in $z_{0}=a^{1/(1-a)}$ with $\phi_{a}\left(z_{0}\right)=z_{0}^{a}$. It holds that*

$$a\cdot z_{0}^{a}\;=\;a^{1+a/(1-a)}\;=\;z_{0}.$$

*Using that $z_{0}^{a}=\exp\left(a\;\ln z_{0}\right)$, this implies that*

$$\left(a\;\ln z_{0}\right)\cdot \exp\left(a\;\ln z_{0}\right)\;=\;z_{0}\;\ln z_{0}.$$

*The Lambert W function is defined to solve the equation wexpw=z as w=W(z), so we get*

$$a\;\ln z_{0}\;=\;W\left(z_{0}\;\ln z_{0}\right)\;\Leftrightarrow \;a\;=\;W\left(z_{0}\;\ln z_{0}\right)/\ln z_{0}.$$

*Thus, since $\phi_{a}\left(z_{0}\right)=a^{a/(1-a)}=a^{1/(1-a)}/a=z_{0}/a$, the dotted curve in Figure 1 follows the function zlnz/W(zlnz). More precisely, since zlnz is minimal in z=1/e with minimal value −1/e, we have to use the principal branch $W\left(z\right)=W_{0}\left(z\right)$ for z≤1/e, and the secondary branch $W\left(z\right)=W_{-1}\left(z\right)$ for z>1/e.*

**Figure 2:**
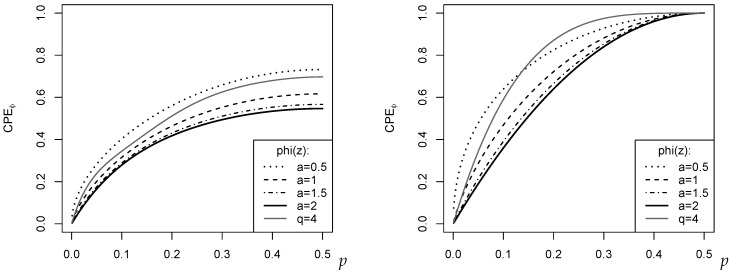
Plot of ${\mathrm{CPE}}_{\phi }$ against *p* for specific cases of $\phi_{a}\left(z\right)=\left(z-z^{a}\right)/\left(a-1\right)$ and $\phi_{q}\left(z\right)=1-{\left|2\;z-1\right|}^{q}$. (**Left**): binomial distribution Bin(4,p); (**Right**): two-point distribution with $f_{0}=p$.

Let us conclude this section with some examples of ${\mathrm{CPE}}_{\phi }$ measures (see Figure 2). For all examples, we set m=4, i.e., we have five ordinal categories like, for example, in the case of a simple Likert scale. In the left part of Figure 2, *f* was computed according to the binomial distribution Bin(4,p), which has maximal dispersion for p=0.5. This is also recognized by any of the plotted measures ${\mathrm{CPE}}_{\phi }$, with their maximal dispersion values varying around 0.6. This medium level of dispersion is plausible because Bin(4,0.5) is far away from the extreme two-point distribution. The right part of Figure 2, by contrast, shows the ${\mathrm{CPE}}_{\phi }$ for the two-point distribution with $f_{0}=p$ ($=f_{1}=\cdots =f_{m-1}$). So p=0 corresponds to a one-point distribution in $s_{m}$ (minimal dispersion), and p=0.5 to the extreme two-point distribution (maximal dispersion). Accordingly, all measures reach their extreme values 0 and 1, respectively. It is interesting to note that outside these extreme cases, the dispersion measures judge the actual dispersion level quite differently; see the related discussion in Kvålseth [10], Weiß [13].

## 3. Asymptotic Distribution of Sample ${\mathrm{CPE}}_{\phi }$

From now on, we focus on the sample version of ${\mathrm{CPE}}_{\phi }$ from (1), i.e., on ${\hat{\mathrm{CPE}}}_{\phi }={\mathrm{CPE}}_{\phi }\left(\hat{f}\right)$, where $\hat{f}$ denotes the vector of cumulative relative frequencies computed from $X_{1},\ldots ,X_{n}$. To derive the asymptotic distribution of ${\hat{\mathrm{CPE}}}_{\phi }$, which is to be used as an approximation to the true distribution of ${\hat{\mathrm{CPE}}}_{\phi }$, we recall the following results from Weiß [14]. Provided that the data-generating process (DGP) satisfies appropriate mixing conditions, e.g., α-mixing with exponentially decreasing weights (which includes the i. i. d.-case), it holds that

(8) $$\begin{array}{c} \sqrt{n}\;\left(\hat{f}-f\right)\;\overset{d}{\to }\;N\left(0,\Sigma \right)\;\mathrm{with}\;\Sigma ={\left(\sigma_{ij}\right)}_{i,j=0,\ldots ,m-1},\;\mathrm{where}\\ \sigma_{ij}\;\overset{a}{=}\;f_{\min\left\{i,j\right\}}-f_{i}\;f_{j}\;+\;\sum_{h=1}^{\infty }\;(f_{ij}\left(h\right)+f_{ji}\left(h\right)-2\;f_{i}\;f_{j}). \end{array}$$

For an analogous result in the presence of missing data, see Theorem 1 in Weiß [15]. In (8), finite (co)variances are ensured if we require that $\sum_{h=1}^{\infty }\left(f_{ij}\left(h\right)-f_{i}\;f_{j}\right)\;<\infty$ holds for all i,j (“short memory”). In particular, all sums $\sum_{h=1}^{\infty }\;\left(f_{ij}\left(h\right)+f_{ji}\left(h\right)-2\;f_{i}\;f_{j}\right)$ vanish in the i. i. d.-case. Otherwise, they account for the serial dependence of the DGP. This can be seen by considering the trace of Σ, which equals

$$\sum_{i=0}^{m-1}\left(f_{i}\left(1-f_{i}\right)+2\;\sum_{h=1}^{\infty }\;\left(f_{ii}\left(h\right)-f_{i}^{2}\right)\right)\;=\;\sum_{i=0}^{m-1}f_{i}\left(1-f_{i}\right)\;\left(1+2\;\sum_{h=1}^{\infty }\kappa_{\mathrm{ord}}\left(h\right)\right).$$

Here, the term $\sum_{i=0}^{m-1}f_{i}\left(1-f_{i}\right)$ agrees with the IOV in (4) except the normalizing factor $\frac{4}{m}$, i.e., it corresponds to ${\mathrm{CPE}}_{\phi }$ with ϕ(z)=z(1−z). The term $\kappa_{\mathrm{ord}}\left(h\right)$, in turn, is the ordinal Cohen’s κ [14] defined by

(9) $$\kappa_{\mathrm{ord}}\left(h\right)\;=\;\frac{\sum_{i=0}^{m-1}\left(f_{ii}\left(h\right)-f_{i}^{2}\right)}{\sum_{i=0}^{m-1}f_{i}\left(1-f_{i}\right)}.$$

It is a measure of signed serial dependence, which evaluates the extent of (dis)agreement between $X_{t}$ and $X_{t-h}$ by positive (negative) values.

Based on Taylor expansions of ${\hat{\mathrm{CPE}}}_{\phi }={\mathrm{CPE}}_{\phi }\left(\hat{f}\right)$ in ***f***, we shall now study its asymptotic behavior. To establish asymptotic normality and to derive the asymptotic variance of ${\hat{\mathrm{CPE}}}_{\phi }$, we need ϕ to be differentiable. For an asymptotic bias correction, which relies on a second-order Taylor expansion, ϕ even has to be twice differentiable (then, the concavity of ϕ implies that $\phi^{\prime \prime }\left(z\right)<0$).

**Remark** **2.** *From the examples discussed in Section 2, the EGF corresponding to the LOV (i.e., $\phi_{q}$ with q=1) is not differentiable (in z=1/2). $\phi_{q}$ is only once differentiable for 1<q<2, while q≥2 ensures $\phi_{q}$ to be at least twice differentiable; see Example 1 below. Accordingly, in these cases, it is not possible to establish asymptotic normality (q=1) or an asymptotic bias correction (1<q<2), respectively. In fact, Weiß [13] was faced with the same problem when studying the asymptotics of the sample ${\mathrm{OV}}_{q}$, and a solution to it was not possible. In simulations, he showed that even modified asymptotics (using a folded-normal distribution) did not lead to an acceptable approximation quality. We shall therefore exclude such cases from our further discussion. If, in applications, the cases q=1 or 1<q<2 appear to be relevant, a bootstrap approach might be an option to gain insight into the sample distribution of ${\hat{\mathrm{CPE}}}_{\phi }$.*

Assuming ϕ to be (twice) differentiable, the partial derivatives of ${\mathrm{CPE}}_{\phi }\left(f\right)$ according to (1) are

(10) $$\begin{array}{cc} \frac{\partial }{\partial f_{k}}\;CPE_{\phi }\left(f\right)\;= & \frac{1}{2\;m\;\phi (1/2)}\;\left(\phi^{\prime }\left(f_{k}\right)-\phi^{\prime }\left(1-f_{k}\right)\right)\;=:d_{k},\\ \frac{\partial^{2}}{\partial^{2}f_{k}}\;CPE_{\phi }\left(f\right)\;= & \frac{1}{2\;m\;\phi (1/2)}\;\left(\phi^{\prime \prime }\left(f_{k}\right)+\phi^{\prime \prime }\left(1-f_{k}\right)\right)\;=:h_{kk},\\ \frac{\partial^{2}}{\partial f_{k}\;\partial f_{l}}\;CPE_{\phi }\left(f\right)\;= & 0\;\mathrm{for}\;k\neq l. \end{array}$$

We denote the gradient (Jacobian) of ${\mathrm{CPE}}_{\phi }\left(f\right)$ by $D=(d_{0},\ldots ,d_{m-1})$, and the Hessian equals $H=diag(h_{00},\ldots ,h_{m-1,m-1})$. Note that if ϕ is symmetric around 1/2, i.e., if ϕ(z)=ϕ(1−z), then $d_{k}=\phi^{\prime }\left(f_{k}\right)/\left(m\;\phi (1/2)\right)$ and $h_{kk}=\phi^{\prime \prime }\left(f_{k}\right)/\left(m\;\phi (1/2)\right)$.

**Example** **1.** *Let us compute the derivatives required in (10) for the EGF examples presented in Section 2.*

*For $\phi_{a}\left(z\right)=\frac{z-z^{a}}{a-1}$, the constant factor becomes $\frac{1}{2\;m\;\phi_{a}\left(1/2\right)}=\frac{2^{a-1}\;\left(a-1\right)}{m\;(2^{a-1}-1)}$, and the derivatives are $\phi_{a}^{\prime }\left(z\right)=\frac{1-a\;z^{a-1}}{a-1}$ and $\phi_{a}^{\prime \prime }\left(z\right)=-a\;z^{a-2}$. Here, $\phi_{a}^{\prime }\left(z\right)$ exists in the boundary value z=0 only if a>1, and $\phi_{a}^{\prime \prime }\left(z\right)$ if a≥2. Important special cases are*

$$\phi \left(z\right)=-z\;\ln z\;\Rightarrow \;\frac{1}{2\;m\;\phi (1/2)}=\frac{1}{m\;\ln2},\;\phi^{\prime }\left(z\right)=-1-\ln z,\;\phi^{\prime \prime }\left(z\right)=-1/z\;\mathrm{for}\;a\to 1,$$

$$\phi \left(z\right)=z\left(1-z\right)\;\Rightarrow \;\frac{1}{2\;m\;\phi (1/2)}=\frac{2}{m},\;\phi^{\prime }\left(z\right)=1-2\;z,\;\phi^{\prime \prime }\left(z\right)=-2\;\mathrm{for}\;a=2,$$

*and for a=3,*

$$\phi \left(z\right)=\frac{1}{2}\;z\;\left(1-z^{2}\right)\;\Rightarrow \;\frac{1}{2\;m\;\phi (1/2)}=\frac{8}{3\;m},\;\phi^{\prime }\left(z\right)=\frac{1}{2}\;\left(1-3\;z^{2}\right),\;\phi^{\prime \prime }\left(z\right)=-3\;z.$$

*Note that both a=2,3 lead to the same expressions for $d_{k},h_{kk}$ in (10); see Table 1. This is in accordance with the equivalence of these cases as discussed after (7).*

*For $\phi_{q}\left(z\right)=1-{\left(1-2\;\min\left\{z,1-z\right\}\right)}^{q}=1-{\left|2\;z-1\right|}^{q}$ with $\frac{1}{2\;m\;\phi_{q}\left(1/2\right)}=\frac{1}{2\;m}$, the derivatives are expressed using the sign function, sgn(·), which is not continuous at 0. Note that the following relations hold:*

$$\frac{d}{dx}\;|x|\;=\;\mathrm{sgn}\left(x\right)\;for\;x\neq 0,\;x\;=\;\mathrm{sgn}\left(x\right)\cdot |x|,\;|x|\;=\;\mathrm{sgn}\left(x\right)\cdot x.$$

*For q≥2, it then follows by applying the chain rule and the product rule that*

$$\begin{array}{cc} \phi_{q}^{\prime }\left(z\right)\;= & {-2\;q\;|2\;z-1|}^{q-1}\;\mathrm{sgn}\left(2\;z-1\right)\;=\;-2\;q\;\left(2\;z-1\right)\;{\left|2\;z-1\right|}^{q-2},\\ \phi_{q}^{\prime \prime }\left(z\right)= & {-4\;q\;|2\;z-1|}^{q-2}\;-\;4\;q\left(q-2\right)\;\left(2\;z-1\right)\;{\left|2\;z-1\right|}^{q-3}\;\mathrm{sgn}\left(2\;z-1\right)\\ = & {-4\;q\left(q-1\right)\;|2\;z-1|}^{q-2}. \end{array}$$

*Note that these derivatives are continuous in z=1/2 for q≥2, using that $0^{0}=1$. The final expressions for (10) are summarized in Table 1.*

**Table 1:** Partial derivatives (10) for EGFs discussed in Example 1.

EGF ϕ(z)	$d_{k}$	$h_{\mathrm{kk}}$
$\left(z-z^{a}\right)/\left(a-1\right)$	$\frac{2^{a-1}\;a}{m\;(2^{a-1}-1)}\;\left({\left(1-f_{k}\right)}^{a-1}-f_{k}^{a-1}\right)$	$-\frac{2^{a-1}\;a\;\left(a-1\right)}{m\;(2^{a-1}-1)}\;\left(f_{k}^{a-2}+{\left(1-f_{k}\right)}^{a-2}\right)$
−zlnz	$\frac{1}{m\;\ln2}\;(\ln\left(1-f_{k}\right)-\ln f_{k})$	$\frac{-1}{m\;\ln2}\;(1/f_{k}+1/\left(1-f_{k}\right))$
$z\left(1-z\right),\;\frac{1}{2}\;z\;\left(1-z^{2}\right)$	$\frac{4}{m}\;\left(1-2\;f_{k}\right)$	$-\frac{8}{m}$
${1-|2\;z-1|}^{q}$	$-\frac{2\;q}{m}\;\left(2\;f_{k}-1\right)\;{\left|2\;f_{k}-1\right|}^{q-2}$	$-\frac{4\;q(q-1)}{m}\;{\left|2\;f_{k}-1\right|}^{q-2}$

Using (10), the second-order Taylor expansion equals

(11) $${\mathrm{CPE}}_{\phi }\left(\hat{f}\right)\;\approx \;{\mathrm{CPE}}_{\phi }\left(f\right)\;+\;\sum_{k=0}^{m-1}d_{k}\;\left({\hat{f}}_{k}-f_{k}\right)\;+\;\frac{1}{2}\;\sum_{k=0}^{m-1}h_{kk}\;{\left({\hat{f}}_{k}-f_{k}\right)}^{2}.$$

According to (8), the linear term in (11) is asymptotically normally distributed (“Delta method”), provided that D does not vanish (see Remark 3 below). Then, we conclude from (8) that

(12) $$\sqrt{n}\;\left({\mathrm{CPE}}_{\phi }\left(\hat{f}\right)-{\mathrm{CPE}}_{\phi }\left(f\right)\right)\;\overset{d}{\to }\;N\left(0,\;D\;\Sigma \;D{}^{⊤}\right),\;D\;\Sigma \;D{}^{⊤}=\sum_{i,j=0}^{m-1}d_{i}\;d_{j}\;\sigma_{ij}.$$

The approximate bias correction relies on the quadratic term in (11), because $E[{\hat{f}}_{k}-f_{k}]=0$. Using (8), we conclude that

(13) $$n\;E\left[{\mathrm{CPE}}_{\phi }(\hat{f})-{\mathrm{CPE}}_{\phi }\left(f\right)\right]\;\approx \;\frac{1}{2}\;\sum_{i=0}^{m-1}h_{ii}\;\sigma_{ii}.$$

Let us summarize the results implied by (12) and (13) in the following theorem.

**Theorem** **1.** *Under the mixing assumptions stated before (8), assuming the EGF ϕ to be twice differentiable, and recalling the $d_{k},h_{kk}$ from (10) where D must not vanish, it holds that*

$$\begin{array}{c} \sqrt{n}\;\left({\mathrm{CPE}}_{\phi }(\hat{f}\right)-{\mathrm{CPE}}_{\phi }\left(f\right))\;\overset{d}{\to }\;N\left(0,\;\sigma_{\phi }^{2}\right),\;\sigma_{\phi }^{2}\;=\;\sigma_{\phi ,iid}^{2}\;\left(1\;+2\;\sum_{h=1}^{\infty }\vartheta_{\phi }\left(h\right)\right)\\ with\;\sigma_{\phi ,iid}^{2}\;=\;\sum_{i,j=0}^{m-1}d_{i}\;d_{j}\;\left(f_{\min\left\{i,j\right\}}-f_{i}\;f_{j}\right)\;and\;\vartheta_{\phi }\left(h\right)\;=\;\frac{\sum_{i,j=0}^{m-1}d_{i}\;d_{j}\;\left(f_{ij}\left(h\right)-f_{i}\;f_{j}\right)}{\sum_{i,j=0}^{m-1}d_{i}\;d_{j}\;\left(f_{\min\left\{i,j\right\}}-f_{i}\;f_{j}\right)}. \end{array}$$

*In addition, the bias-corrected mean of ${\mathrm{CPE}}_{\phi }(\hat{f})$ is*

$$\begin{array}{c} E\left[{\mathrm{CPE}}_{\phi }\left(\hat{f}\right)\right]\;\approx \;{\mathrm{CPE}}_{\phi }\left(f\right)\;+\;\frac{1}{2\;n}\;\left(\sum_{i=0}^{m-1}h_{ii}\;f_{i}\left(1-f_{i}\right)\right)\;\left(1\;+2\;\sum_{h=1}^{\infty }\kappa_{\phi }\left(h\right)\right),\\ where\;\kappa_{\phi }\left(h\right)\;=\;\frac{\sum_{i=0}^{m-1}h_{ii}\;\left(f_{ii}\left(h\right)-f_{i}^{2}\right)}{\sum_{i=0}^{m-1}h_{ii}\;f_{i}\left(1-f_{i}\right)}. \end{array}$$

Note that the second-order derivatives are negative due to the concavity of ϕ, so ${\mathrm{CPE}}_{\phi }(\hat{f})$ exhibits a negative bias. $\vartheta_{\phi }\left(h\right)$ expresses the effect of serial dependence on $\sigma_{\phi }^{2}$. For i. i. d. ordinal data, $\vartheta_{\phi }\left(h\right)=0$, so Theorem 1 simplifies considerably in this case, namely to $\sigma_{\phi }^{2}=\sigma_{\phi ,iid}^{2}$. The bias of ${\mathrm{CPE}}_{\phi }(\hat{f})$ is affected by serial dependence via $\kappa_{\phi }\left(h\right)$, which is a κ-type measure reflecting the extent of (dis)agreement between lagged observations, recall (9). More precisely, $\kappa_{\phi }\left(h\right)$ can be interpreted as weighted type of $\kappa_{\mathrm{ord}}\left(h\right)$, where the weights $h_{ii}$ depend on the particular choice of ϕ. It thus provides a novel family of measures of signed serial dependence, the asymptotics of which are analyzed in Section 4 below.

**Example** **2.** *In the special case ϕ(z)=z(1−z) (as well as for $\phi \left(z\right)=\frac{1}{2}\;z\;\left(1-z^{2}\right)$), which corresponds to the IOV in (4), $h_{ii}=-\frac{8}{m}$ is constant (see Table 1). Thus, $\kappa_{\phi }\left(h\right)=\kappa_{\mathrm{ord}}\left(h\right)$ in this case (see (9)). Furthermore, the first factor of the bias in Theorem 1 becomes*

$$\frac{1}{2\;n}\;\sum_{i=0}^{m-1}h_{ii}\;f_{i}\left(1-f_{i}\right)\;=\;-\frac{1}{n}\cdot \frac{4}{m}\sum_{i=0}^{m-1}f_{i}\left(1-f_{i}\right)\;=\;-\frac{1}{n}\;\mathrm{IOV}.$$

*Hence, the bias is determined by both the serial dependence and the dispersion of the process.*

*As another simple example, consider the choice ϕ(z)=−zlnz for the CPE in (5). Then, using $h_{ii}=\frac{-1}{m\;\ln2}\;\left(\frac{1}{f_{i}}+\frac{1}{1-f_{i}}\right)=\frac{-1}{m\;\ln2}\;\frac{1}{f_{i}\left(1-f_{i}\right)}$ from Table 1, we get*

$$\frac{1}{2\;n}\;\sum_{i=0}^{m-1}h_{ii}\;f_{i}\left(1-f_{i}\right)\;=\;\frac{1}{2\;n}\;\frac{-1}{m\;\ln2}\;\sum_{i=0}^{m-1}1\;=\;\frac{-1}{(2\;\ln2)\;n}.$$

*Thus, we have a unique i. i. d.-bias, independent of the actual marginal CDF **f**. Under serial dependence, we get*

(14) $$\kappa_{\phi }\left(h\right)\;=\;\frac{\sum_{i=0}^{m-1}h_{ii}\;\left(f_{ii}\left(h\right)-f_{i}^{2}\right)}{\sum_{i=0}^{m-1}h_{ii}\;f_{i}\left(1-f_{i}\right)}\;=\;\frac{1}{m}\;\sum_{i=0}^{m-1}\frac{f_{ii}\left(h\right)-f_{i}^{2}}{f_{i}\left(1-f_{i}\right)}\;=:\;\kappa_{ord}^{*}\left(h\right).$$

So, besides the pair $\left(\mathrm{IOV},\kappa_{\mathrm{ord}}\left(h\right)\right)$, $\left(\mathrm{CPE},\kappa_{\mathrm{ord}}^{*}\left(h\right)\right)$ also belongs to the $\left({\mathrm{CPE}}_{\phi },\kappa_{\phi }\left(h\right)\right)$-family.

**Figure 3:**
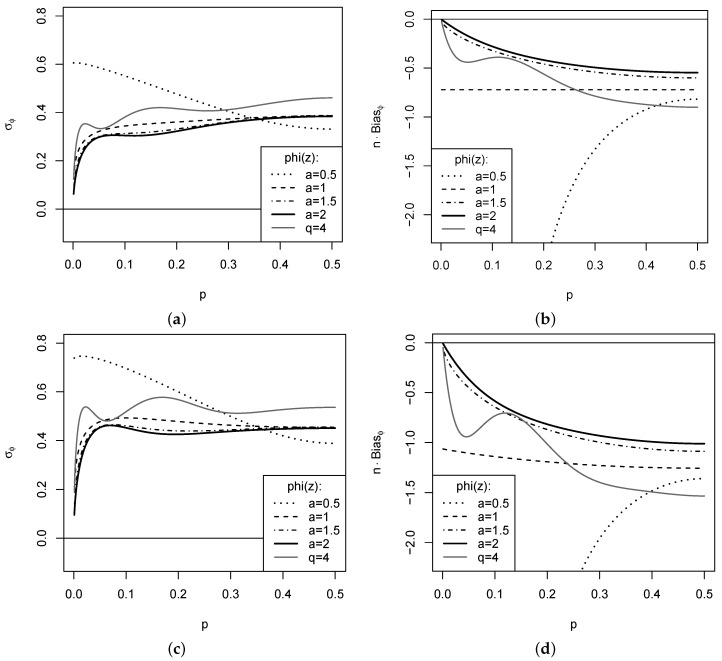
Plots for specific cases of $\phi_{a}\left(z\right)=\left(z-z^{a}\right)/\left(a-1\right)$ and $\phi_{q}\left(z\right)=1-{\left|2\;z-1\right|}^{q}$: $\sigma_{\phi }$ (left) and $n\;(E[CPE_{\phi }(\hat{f})]-CPE_{\phi }\left(f\right))$ (right) against *p* of marginal Bin(4,p), where i. i. d. DGP in (**a**,**b**), and BAR(1) DGP with dependence parameter ρ=0.4 in (**c**,**d**).

*A few examples are plotted in Figure 3, where the DGP $X_{t}=s_{I_{t}}$ assumes the rank counts $I_{t}$ to have Bin(4,p)-marginals. In the top panel, the DGP is i. i. d., whereas $\left(I_{t}\right)$ follows a so-called first-order binomial autoregressive (BAR(1)) model with dependence parameter ρ=0.4 ([2] Section 3.3) in the lower panel, i.e., the DGP exhibits a medium level of positive dependence. While the resulting dependence structure is investigated in more detail in Section 4, Figure 3 considers the asymptotic standard error (SE) $\sigma_{\phi }$ and bias $n\;(E[{\mathrm{CPE}}_{\phi }(\hat{f})]-{\mathrm{CPE}}_{\phi }\left(f\right))$ according to Theorem 1. Obviously, an increase of serial dependence causes an increase of both SE and bias. While most measures have a rather stable SE for varying p (except for extremely small p, where we are close to a one-point distribution), the EGF $\phi_{a}$ with a=1/2 varies a lot. In particular, the bias takes rather extreme values with decreasing p for this case, which can be explained by the strongly negative exponents at $f_{k},1-f_{k}$ in $h_{kk}$ from Table 1. Thus, choices a<1 seem not advisable for practice. The boundary case a=1 has a constant bias for an i. i. d. DGP. For $\phi_{q}$ with q=4, we note an oscillating behavior of both bias and SE. The lowest bias and SE are achieved for the cases $\phi_{a}$ with a>1.*

The newly obtained measure $\kappa_{\mathrm{ord}}^{*}\left(h\right)$ from (14) constitutes a counterpart to the nominal measures $\kappa^{*}\left(h\right),\kappa^{☆}\left(h\right)$ in Weiß [20]. It is worth pointing out that the latter measures were derived from the nominal entropy and extropy, respectively, while the CPE in (5) can be interpreted as a combination of cumulative entropy and extropy. It has to be noted that $\kappa_{\mathrm{ord}}^{*}\left(h\right)$ also shares a disadvantage with $\kappa^{*}\left(h\right)$: if only one of the $f_{i}$ equals 0 or 1, we suffer from a division by 0 in (14). For $\kappa_{\mathrm{ord}}\left(h\right)$ according to (9), by contrast, a division by 0 only happens in the (deterministic) case of a one-point distribution. As a workaround when computing $\kappa_{\mathrm{ord}}^{*}\left(h\right)$, it is recommended to replace the affected summands in (14) by 0.

**Remark** **3.** *If $f=f_{two}$, then all $d_{k}=0$ in (10). Therefore, the linear term in (11) vanishes. In fact, for any two-point distribution on $s_{0}$ and $s_{m}$, we necessarily have $f_{0}=\ldots =f_{m-1}$ and ${\hat{f}}_{0}=\ldots ={\hat{f}}_{m-1}$. Therefore, ${\mathrm{CPE}}_{\phi }(\hat{f})$ reduces to ${\mathrm{CPE}}_{\phi }(\hat{f})=\frac{1}{2\;\phi (1/2)}\;(\phi \left({\hat{f}}_{0}\right)+\phi (1-{\hat{f}}_{0}))$, and the quadratic term in (11) to $\frac{m}{2}\;h_{00}\;{\left({\hat{f}}_{0}-f_{0}\right)}^{2}$. Hence, in this special case,*

$$n\;({\mathrm{CPE}}_{\phi }(\hat{f})-{\mathrm{CPE}}_{\phi }\left(f\right))\;\overset{a}{∼}\;\frac{m}{2}\;h_{00}\;\sigma_{00}\cdot \chi_{1}^{2}.$$

*For example, plugging-in $h_{00}=-\frac{8}{m}$ for ϕ(z)=z(1−z) corresponding to the IOV in (4), we obtain the result in Remark 7.1.2 in Weiß* [14].

## 4. Asymptotic Distribution of Sample $\kappa_{\phi }\left(h\right)$

**Figure 4:**
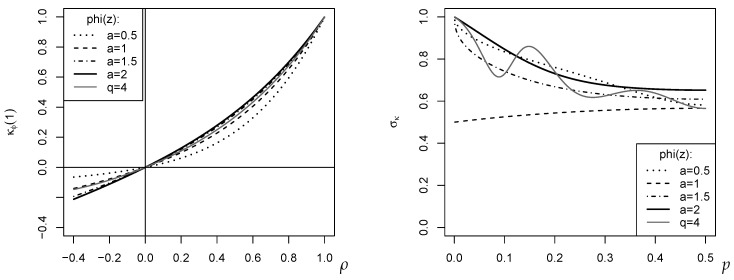
Plots for specific cases of $\phi_{a}\left(z\right)=\left(z-z^{a}\right)/\left(a-1\right)$ and $\phi_{q}\left(z\right)=1-{\left|2\;z-1\right|}^{q}$: $\kappa_{\phi }\left(1\right)$ against BAR(1)’s dependence parameter ρ with marginal Bin(4,0.3) (**left**); $\sigma_{\kappa }$ against *p* of marginal Bin(4,0.3) (**right**).

The bias equation in Theorem 1 gives rise to a novel family of serial dependence measures for ordinal time series, namely

(15) $$\kappa_{\phi }\left(h\right)\;=\;\frac{\sum_{i=0}^{m-1}\left(\phi^{\prime \prime }\left(f_{i}\right)+\phi^{\prime \prime }\left(1-f_{i}\right)\right)\;\left(f_{ii}\left(h\right)-f_{i}^{2}\right)}{\sum_{i=0}^{m-1}\left(\phi^{\prime \prime }\left(f_{i}\right)+\phi^{\prime \prime }\left(1-f_{i}\right)\right)\;f_{i}\left(1-f_{i}\right)}$$

for a given EGF ϕ. Some examples are plotted in the left part of Figure 4, where the DGP $X_{t}=s_{I_{t}}$ assumes that the rank counts $I_{t}$ follow the BAR(1) model with marginal distribution Bin(4,0.3) and dependence parameter ρ; recall the discussion of Figure 3. So, the rank counts ${\left(I_{t}\right)}_{Z}$ have the first-order ACF ρ, whereas the plotted $\kappa_{\phi }\left(1\right)$ have absolute value ≤|ρ|.

In practice, the sample version of this measure, ${\hat{\kappa }}_{\phi }\left(h\right)$, is particularly important, where the cumulative (bivariate) probabilities are replaced by the corresponding relative frequencies. For uncovering significant deviations from the null hypothesis of serial independence (then, $\kappa_{\phi }\left(h\right)=0$), the asymptotic distribution of ${\hat{\kappa }}_{\phi }\left(h\right)$ under the null of i. i. d. time series data is required. For its derivation, we proceed in an analogous way as in Section 3. As the starting point, we have to extend the asymptotics of the marginal sample CDF in (8) by also considering the bivariate sample CDF ${\hat{f}}_{ii}\left(h\right)$. Let $f_{\left(h\right)}={\left(f_{0},\ldots ,f_{m-1},\;f_{00}\left(h\right),\ldots ,f_{m-1,m-1}\left(h\right)\right)}^{⊤}$, and denote its sample version by ${\hat{f}}_{\left(h\right)}$. Then, under the same mixing conditions as in Section 3, Weiß [14] established the asymptotic normality

(16) $$\sqrt{n}\;\left({\hat{f}}_{\left(h\right)}-f_{\left(h\right)}\right)\;\overset{d}{\to }\;N\left(0,\Sigma^{\left(h\right)}\right)\;\mathrm{with}\;\Sigma^{\left(h\right)}={\left(\sigma_{i,j}^{\left(h\right)}\right)}_{i,j=0,\ldots ,2m-1},$$

and he derived general expressions for the asymptotic (co)variances $\sigma_{i,j}^{\left(h\right)}$. Analogous results for the case of missing data can be found in Supplement S.3 of Weiß [15]. For the present task, the asymptotics of the i. i. d.-case are sufficient. Then, $f_{ii}\left(h\right)=f_{i}^{2}$ for all i=0,…,m−1, and the covariances in (16) are given by

(17) $$\begin{array}{cc} \sigma_{i,j}^{\left(h\right)}=\sigma_{i,j}\;= & f_{\min\left\{i,j\right\}}-f_{i}\;f_{j}\;(\mathrm{see}\;(8)),\\ \sigma_{i,m+j}^{\left(h\right)}\;= & 2\;f_{j}\;\left(f_{\min\left\{i,j\right\}}-f_{i}\;f_{j}\right),\\ \sigma_{m+i,m+j}^{\left(h\right)}\;= & \left(f_{\min\left\{i,j\right\}}+3\;f_{i}\;f_{j}\right)\;\left(f_{\min\left\{i,j\right\}}-f_{i}\;f_{j}\right) \end{array}\mathrm{for}\;i,j\in \left\{0,\ldots ,m-1\right\},$$

see Weiß [14] (as well as p. 8 in Supplement S.3 of Weiß [15] if being concerned with missing data).

Next, we derive the asymptotics of ${\hat{\kappa }}_{\phi }\left(h\right)$ under the i. i. d.-null, and this requires to derive the second-order Taylor expansion for ${\hat{\kappa }}_{\phi }\left(h\right)$; details are postponed to Appendix A.1. As higher-order derivatives of ϕ, which are initially used while deriving a bias correction of ${\hat{\kappa }}_{\phi }\left(h\right)$, cancel out, the final result still relies on derivatives of ϕ up to order 2 only.

**Theorem** **2.** *Under the null hypothesis of i. i. d. data, i.e., if $\kappa_{\phi }\left(h\right)=0$ for all lags h∈N, and assuming the EGF ϕ to be twice differentiable, it holds that*

$$\begin{array}{c} \sqrt{n}\;\left({\hat{\kappa }}_{\phi }\left(h\right)-\kappa_{\phi }\left(h\right)\right)\;\overset{d}{\to }\;N\left(0,\;\sigma_{\kappa }^{2}\right)\;with\;\sigma_{\kappa }^{2}\;=\;\sum_{j,k=0}^{m-1}u_{j}\;u_{k}\;{\left(f_{\min\left\{j,k\right\}}-f_{j}\;f_{k}\right)}^{2},\\ where\;u_{j}\;=\;\frac{\phi^{\prime \prime }\left(f_{j}\right)+\phi^{\prime \prime }\left(1-f_{j}\right)}{\sum_{i=0}^{m-1}\left(\phi^{\prime \prime }\left(f_{i}\right)+\phi^{\prime \prime }\left(1-f_{i}\right)\right)\;f_{i}\left(1-f_{i}\right)}. \end{array}$$

*In addition, the bias-corrected mean of ${\hat{\kappa }}_{\phi }\left(h\right)$ is $E\left[{\hat{\kappa }}_{\phi }\left(h\right)\right]\;\approx \;-\frac{1}{n}$.*

Note that we have a unique bias correction for any of the measures ${\hat{\kappa }}_{\phi }\left(h\right)$, independent of the choice of the EGF ϕ. Thus, for application in practice, it remains to compute the asymptotic variance $\sigma_{\kappa }^{2}$ in Theorem 2. This only requires knowledge about $\phi^{\prime \prime }\left(z\right)$ to evaluate the $u_{j}$, but not about higher-order derivatives of the EGF ϕ (see Example 3 for illustration). Further examples are plotted in the right part of Figure 4, where $\sigma_{\kappa }$ was computed for the marginal distribution Bin(4,p). The oscillating behavior of $\sigma_{\kappa }$ for $\phi_{q}\left(z\right)$ with q=4 is quite striking. It is also interesting to note that among the plotted κ-measures, the novel ${\hat{\kappa }}_{\mathrm{ord}}^{*}\left(h\right)$ (case a=1) has the lowest variance.

**Example** **3.** *While we have a unique bias correction for ${\hat{\kappa }}_{\phi }\left(h\right)$, the asymptotic variance $\sigma_{\kappa }^{2}$ according to Theorem 2 differs for different choices of the EGF ϕ, as the involved $u_{j}$ depend on $\phi^{\prime \prime }\left(z\right)$. For example,*

- *for $\phi_{a}\left(z\right)=\frac{z-z^{a}}{a-1}$, we have $\phi_{a}^{\prime \prime }\left(z\right)=-a\;z^{a-2}$ according to Example 1,*
- *while for $\phi_{q}\left(z\right)=1-{\left|2\;z-1\right|}^{q}$ with q≥2, we have $\phi_{q}^{\prime \prime }\left(z\right)=-4\;q\left(q-1\right)\;{\left|2\;z-1\right|}^{q-2}$.*

*Important special cases are:*

- *For ϕ(z)=z(1−z), i.e., for the basic $\kappa_{ord}\left(h\right)$ according to (9), we have*
$$\phi^{\prime \prime }\left(z\right)=-2,\;\mathrm{so}\;u_{j}\;=\;{\left(\sum_{i=0}^{m-1}f_{i}\left(1-f_{i}\right)\right)}^{-1}.$$
*Thus, we get*
$$\sigma_{\kappa }^{2}\;=\;\frac{\sum_{j,k=0}^{m-1}{\left(f_{\min\left\{j,k\right\}}-f_{j}\;f_{k}\right)}^{2}}{{\left(\sum_{i=0}^{m-1}f_{i}\left(1-f_{i}\right)\right)}^{2}};$$
*see Theorem 7.2.1 in Weiß [14].*
- For ϕ(z)=−zlnz, i.e., for the novel $\kappa_{ord}^{*}\left(h\right)$ according to (14), we have
$$\phi^{\prime \prime }\left(z\right)=-\frac{1}{z},\;\mathrm{so}\;u_{j}\;=\;\frac{1}{m}\;\frac{1}{f_{j}\left(1-f_{j}\right)}.$$
*Thus, we get*
$$\sigma_{\kappa }^{2}\;=\;\frac{1}{m^{2}}\;\sum_{j,k=0}^{m-1}\frac{{\left(f_{\min\left\{j,k\right\}}-f_{j}\;f_{k}\right)}^{2}}{f_{j}\left(1-f_{j}\right)\;f_{k}\left(1-f_{k}\right)}\;=\;\frac{1}{m}\;+\;\frac{2}{m^{2}}\;\sum_{j<k}\frac{f_{j}\;\left(1-f_{k}\right)}{f_{k}\;\left(1-f_{j}\right)}.$$

*For any other choice of $\phi_{a}\left(z\right)$ and $\phi_{q}\left(z\right)$, $\sigma_{\kappa }^{2}$ is easily computed using the aforementioned expressions for $\phi_{a}^{\prime \prime }\left(z\right)$ and $\phi_{q}^{\prime \prime }\left(z\right)$ from Example 1. Since the obtained closed-form formulae do not much simplify, further details are omitted here.*

## 5. Simulation Results

In what follows, we discuss results from a simulation study, being tabulated in Appendix B, where $10^{4}$ replications per scenario were used throughout. In view of our previous findings, achieved when discussing the asymptotics plotted in Figure 3 and Figure 4, we do not further consider the choice a=1/2<1 for the EGF $\phi_{a}$, but we use a=5/2>2 instead. The latter choice, in turn, was not presented before as the resulting asymptotic curves could hardly be distinguished from the case a=2. So, altogether, a=1,3/2,2,5/2 as well as q=4 were taken into account for simulations. The ordinal data were generated via binomial rank counts, $X_{t}=s_{I_{t}}$ with $I_{t}∼\mathrm{Bin}\left(m,p\right)$, which either exhibit serial dependence caused by a BAR(1) DGP with dependence parameter ρ, or which are i. i. d. (corresponding to ρ=0).

Let us start with the ordinal dispersion measures ${\hat{\mathrm{CPE}}}_{\phi }$. Table A1 presents the simulated means (upper part) and SEs (lower part) for the case of i. i. d. ordinal data, and these are compared to the asymptotic values obtained from Theorem 1. Generally, we have an excellent agreement between simulated and asymptotic values, i.e., the derived asymptotic approximation to the true distribution of ${\hat{\mathrm{CPE}}}_{\phi }$ works well in practice. This is even more remarkable as also the low sample size n=50 is included. There is a somewhat larger deviation only for the mean in the case a=1, i.e., for the CPE (5), if n≤100 and p=0.1. In this specific case, the simulated sample distribution might be quite close to a one-point distribution, which might cause computational issues for (5); recall that the convention 0ln0=0 has to be used. However, as the approximation quality is good throughout, a pivotal argument for the choice of ϕ in practice might be that the least SEs are observed if using $\phi_{a}$ with a=3/2,2,5/2.

Table A2 considers exactly the same marginals as before, but now in the presence of additional serial dependence (ρ=0.4). Comparing Table A1 and Table A2, it becomes clear that the additional dependence causes increased bias and SE. However, and this is the crucial point for practice, the asymptotic approximations from Theorem 1 work as well as they do in the i. i. d.-case. If there are visible deviations at all, then these happen again mainly for p=0.1 and low sample sizes. Overall, we have an excellent approximation quality throughout, but with least SEs again for a=3/2,2,5/2.

While the ${\mathrm{CPE}}_{\phi }$-type dispersion measures and their asymptotics perform well, essentially for any choice of ϕ, the gap becomes wider when looking at the serial dependence measure ${\hat{\kappa }}_{\phi }\left(h\right)$. The asymptotics in Theorem 2 refer to the i. i. d.-case, which is used as the null hypothesis ($H_{0}$) if testing for significant serial dependence. Thus, let us start by investigating again the mean and SE of ${\hat{\kappa }}_{\phi }\left(1\right)$ for i. i. d. data (same DGPs as in Table A1); see the results in Table A3. For the asymptotic mean, we have the unique approximation −1/n, and this works well except for p=0.1 and low sample sizes. In particular, for $\phi_{a}$ with a=1, i.e., for ${\hat{\kappa }}_{\mathrm{ord}}^{*}\left(1\right)$, we get notable deviations. The reason is given by the computation of ${\hat{\kappa }}_{\mathrm{ord}}^{*}\left(1\right)$, where division by zero might happen (in the simulations, this was circumvented by replacing a zero by $10^{-6}$). In a weakened form, we observe a similar issue for the case a=3/2; generally, we are faced with the zero problem if a<2 because of the second-order derivatives of $\phi_{a}\left(z\right)$. Analogous deviations are observed for the SEs. Here, generally, the simulated SEs tend to be larger than the asymptotic ones. As a consequence, if using the asymptotic SEs for calculating the critical values when testing $H_{0}$, we expect a tendency to oversizing.

If looking at the simulated rejection rates in Table A4, first at the size values (ρ=0) being printed in italic font, we indeed see sizes being slightly larger than the nominal 5%-level, as long as n≤100. For larger sample sizes, by contrast, the ${\hat{\kappa }}_{\phi }\left(1\right)$-test satisfies the given size requirement quite precisely. Here, we computed the critical values by plugging-in the respective sample CDF $\hat{f}$ into the formula for $\sigma_{\kappa }^{2}$. In Table A4, power values for ρ≠0 are also shown. Note that for a BAR(1) process, ρ can take any positive value in (0;1), but the attainable range of negative values is bounded from below by $\max\left\{\;-\frac{1-p}{p},-\frac{p}{1-p}\right\}$ [2]. Thus, only ρ=−0.4,−0.2 are considered in Table A4. Generally, the ${\hat{\kappa }}_{\phi }\left(1\right)$-tests are powerful regarding both positive and negative dependencies, but the actual power performance differs a lot for different ϕ. The worst power is observed for $\phi_{q}$ with q=4, followed by $\phi_{a}$ with a=1. Regarding the remaining $\phi_{a}$-cases, a=3/2 does slightly worse than a=2,5/2, and we often have a slight advantage for a=5/2, especially for negative dependencies.

To sum up, while the whole $\left({\mathrm{CPE}}_{\phi },\kappa_{\phi }\left(h\right)\right)$-family is theoretically appealing, and while there are hardly any noteworthy problems with the sample dispersion measures ${\hat{\mathrm{CPE}}}_{\phi }$, the performance of ${\hat{\kappa }}_{\phi }\left(h\right)$ clearly depends on the choice of ϕ. It is recommended to use the family of *a*-entropies (6), and there, a≥2 is preferable. The measure ${\hat{\kappa }}_{\mathrm{ord}}^{*}\left(1\right)$ from (14), for example, although theoretically appealing as a combination of entropy and extropy, has a relatively bad finite-sample performance. The probably most well-known pair, $\left(\mathrm{IOV},\kappa_{\mathrm{ord}}\left(h\right)\right)$, has a good performance, although there appears to be a slight advantage if choosing *a* somewhat larger than 2, such as a=5/2 (recall that a=3 leads back to the case a=2).

## 6. Data Application

Ordinal time series are observed in quite diverse application areas. Economic examples include time series on credit ratings [14] or on fear states at the stock market [20], and a climatological example is the level of cloudiness of the sky [23]. Health-related examples are time series of electroencephalographic (EEG) sleep states [24], the pain severity of migraine attacks, and the level of perceived stress [15]. In this section, we are concerned with an environmental application, namely the level of air quality. Different definitions of air quality have been reported in the literature. In Chen Chiu [25], the air quality index (AQI) is used for expressing the daily air quality, with levels ranging from $s_{0}=“\mathrm{good}”$ to $s_{5}=“\mathrm{hazardous}”$. Another case study is reported by Liu et al. [26], who use the classification of the Chinese government, which again distinguishes m+1=6 levels, but now ranging from $s_{0}=“\mathrm{excellent}”$ to $s_{5}=“\mathrm{severely}\;\mathrm{polluted}”$. The latter article investigates daily time series from thirty Chinese cities for the period December 2013–July 2019, i.e., the sample size equals n=2068. In what follows, we use one of the time series studied by Liu et al. [26], namely the daily air quality levels $x_{1},\ldots ,x_{n}$ in Shanghai, for illustrating our novel results about cumulative paired ϕ-entropies.

**Figure 5:**
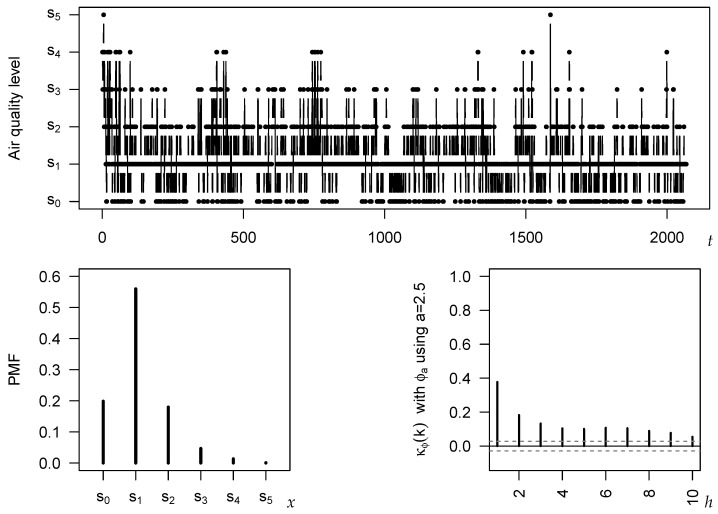
Daily air quality level in Shanghai: plot of time series $\left(x_{t}\right)$ in top panel; plot of sample PMF (**left**) and ${\hat{\kappa }}_{\phi }\left(h\right)$ (**right**) in bottom panel; ${\hat{\kappa }}_{\phi }\left(h\right)$ uses *a*-entropy with a=5/2.

The considered time series is plotted in the top panel of Figure 5. The bottom left graph shows the sample version of the probability mass function (PMF) $P(X=s_{i})$, i.e., the relative frequencies of the categories. It exhibits a unimodal shape with mode (=median) in $s_{1}=“\mathrm{good}”$. The serial dependence structure is analyzed in the bottom right graph, where ${\hat{\kappa }}_{\phi }\left(h\right)$ with *a*-entropy having a=5/2 is used, as this is the recommended choice according to Section 5. All of the plotted ${\hat{\kappa }}_{\phi }\left(h\right)$-values are significantly different from 0 at the 5 %-level, where the critical values (plotted as dashed lines in Figure 5) are computed as {−0.029,0.028} according to Theorem 2 (and by plugging-in the sample CDF). We recognize a medium level of dependence (${\hat{\kappa }}_{\phi }\left(1\right)\approx 0.378$), which quickly decreases with increasing time lag *h*, similar to an AR-type process.

Let us now have a closer look at the dispersion properties of the Shanghai series. The different choices of the ${\mathrm{CPE}}_{\phi }$-measure considered so far provide slightly different results regarding the extent of dispersion. In accordance with Figure 2, the largest point estimates are computed for a=1/2 (0.514) and q=4 (0.465), followed by a=1 with 0.394, whereas a=3/2 (0.349), a=2 (0.332), and a=5/2 (0.328) lead to similar but clearly lower values. Comparing the sample PMF in Figure 5 to the extreme scenarios of a one-point and an extreme two-point distribution, the PMF appears to be more close to a one-point than to a two-point distribution, i.e., the lower ones among the above dispersion values seem to be more realistic here.

The novel asymptotics of Theorem 1 allow to judge the estimation uncertainty for the above point estimates. To keep the discussion simple, let us focus again on the case a=5/2. In the first step, we compute the i. i. d.-approximations of bias and SE, $\frac{1}{2\;n}\;(\sum_{i=0}^{m-1}h_{ii}\;f_{i}\left(1-f_{i}\right))$ and $n^{-1/2}\;\sigma_{\phi ,iid}$, respectively. By plugging-in the sample CDF, these are obtained as $-1.54\cdot 10^{-4}$ and $7.83\cdot 10^{-3}$, respectively. However, these i. i. d.-results are misleading in the present example as the data exhibit significant serial dependence (recall Figure 5). As we know from Theorem 1, the bias has to be increased by the factor $1+2\sum_{h=1}^{\infty }\kappa_{\phi }\left(h\right)$, and the SE by $\left(1+2\sum_{h=1}^{\infty }\vartheta_{\phi }\left(h\right)\right){}^{1/2}$. These factors shall now be computed based on the so-called “ZOBPAR model” proposed by Liu et al. [26], which constitutes a rank-count approach, $X_{t}=s_{I_{t}}$. In view of the AR(1)-like dependence structure and the high frequency for $s_{1}$, namely 0.560, the conditional distribution of $I_{t}|I_{t-1},\ldots$ is assumed to be a truncated Poisson distribution, truncated to the range {0,…,5}, with time-varying Poisson parameter $\lambda_{t}=0.3489+0.7594\;I_{t-1}$ and additional one-inflation parameter 0.3463 ([26] Table III). For this model fit, we compute

$$1+2\sum_{h=1}^{\infty }\kappa_{\phi }\left(h\right)\;\approx \;1.907,\;\sqrt{1+2\sum_{h=1}^{\infty }\vartheta_{\phi }\left(h\right)}\;\approx \;1.343.$$

Thus, an approximate 95 %-confidence interval (CI) for ${\mathrm{CPE}}_{\phi }$ is given by ≈(0.308;0.349). CIs for the remaining ${\mathrm{CPE}}_{\phi }$-measures are computed analogously, leading to (0.487;0.544) for a=1/2, to (0.372;0.418) for a=1, to (0.328;0.371) for a=3/2, to (0.312;0.353) for a=2, and to (0.439;0.492) for q=4.

## 7. Conclusions

In this article, we considered the family of cumulative paired ϕ-entropies. For each appropriate choice of the EGF ϕ, an ordinal dispersion measure ${\mathrm{CPE}}_{\phi }\left(f\right)$ is implied. For example, particular choices from the families of *a*-entropies or *q*-entropies, respectively, lead to well-known dispersion measures from the literature. The first main contribution of this work was the derivation of the asymptotic distribution of the sample version ${\hat{\mathrm{CPE}}}_{\phi }$ for ordinal time series data. These asymptotics can be used to approximate the true distribution of ${\hat{\mathrm{CPE}}}_{\phi }$, e.g., to compute approximate confidence intervals. Simulations showed that these asymptotics lead to an excellent finite-sample performance. Based on the obtained expression for the asymptotic bias of ${\hat{\mathrm{CPE}}}_{\phi }$, we recognized that each EGF ϕ also implies a κ-type serial dependence measures, i.e., altogether, we have a matched pair $\left({\mathrm{CPE}}_{\phi },\kappa_{\phi }\left(h\right)\right)$ for each EGF ϕ. Again, we analyzed the asymptotics of the sample version ${\hat{\kappa }}_{\phi }\left(h\right)$, and these can be utilized for testing for significant serial dependence in the given ordinal time series. This time, however, the finite-sample performance clearly depends on the choice of ϕ. Choosing ${\hat{\kappa }}_{\phi }\left(h\right)$ based on an *a*-entropy with a≥2, such as a=5/2, ensures good finite-sample properties. The practical application of the measures $\left({\mathrm{CPE}}_{\phi },\kappa_{\phi }\left(h\right)\right)$ and their asymptotics was demonstrated with an ordinal time series on the daily level of air quality in Shanghai.

## Data Availability

The data discussed in Section 6 were taken from Liu et al. [26] and are available at https://doi.org/10.1111/jtsa.12625 (accessed on 22 December 2021).

## Appendix A. Proofs

### Appendix A.1. Proof of Theorem 2

First, we derive the second-order Taylor expansion for ${\hat{\kappa }}_{\phi }\left(h\right)$. For this purpose, define $g:{\left(0;1\right)}^{2\;m}\to R$ by mapping $y=(y_{0},\ldots ,y_{2\;m-1})$ onto

$$g\left(y\right)\;=\;\frac{\sum_{i=0}^{m-1}\left(\phi^{\prime \prime }\left(y_{i}\right)+\phi^{\prime \prime }\left(1-y_{i}\right)\right)\;\left(y_{m+i}-y_{i}^{2}\right)}{\sum_{i=0}^{m-1}\left(\phi^{\prime \prime }\left(y_{i}\right)+\phi^{\prime \prime }\left(1-y_{i}\right)\right)\;y_{i}\left(1-y_{i}\right)}.$$

So, according to (15), $g\left(f_{\left(h\right)}\right)=\kappa_{\phi }\left(h\right)$ and $g\left({\hat{f}}_{\left(h\right)}\right)={\hat{\kappa }}_{\phi }\left(h\right)$. In fact, because of the i. i. d.-assumption, we even have $g\left(f_{\left(h\right)}\right)=0$.

For the intended Taylor expansion of ${\hat{\kappa }}_{\phi }\left(h\right)$, we have to compute all partial derivatives of g(y) up to order 2, and to evaluate these derivatives in $y=f_{\left(h\right)}$, using that $f_{ii}\left(h\right)=f_{i}^{2}$ for all i=0,…,m−1 under the i. i. d.-assumption, and thus $g\left(f_{\left(h\right)}\right)=0$. We use the notations

(A1) $$u_{j}\;:=\;\frac{\phi^{\prime \prime }\left(f_{j}\right)+\phi^{\prime \prime }\left(1-f_{j}\right)}{\sum_{i=0}^{m-1}\left(\phi^{\prime \prime }\left(f_{i}\right)+\phi^{\prime \prime }\left(1-f_{i}\right)\right)\;f_{i}\left(1-f_{i}\right)},\;v_{j}\;:=\;\frac{\phi^{\prime \prime \prime }\left(f_{j}\right)-\phi^{\prime \prime \prime }\left(1-f_{j}\right)}{\sum_{i=0}^{m-1}\left(\phi^{\prime \prime }\left(f_{i}\right)+\phi^{\prime \prime }\left(1-f_{i}\right)\right)\;f_{i}\left(1-f_{i}\right)}.$$

Note that if ϕ is symmetric around 1/2, i.e., if ϕ(z)=ϕ(1−z), then $u_{j},v_{j}$ simplify because of $\phi^{\prime \prime }\left(f_{j}\right)+\phi^{\prime \prime }\left(1-f_{j}\right)=2\;\phi^{\prime \prime }\left(f_{j}\right)$ and $\phi^{\prime \prime \prime }\left(f_{j}\right)-\phi^{\prime \prime \prime }\left(1-f_{j}\right)=2\;\phi^{\prime \prime \prime }\left(f_{j}\right)$. Using (A1), we get for 0≤j,k≤m−1 that

(A2) $$\begin{array}{l} d_{j}^{\left(\kappa \right)}\;:=\;\frac{\partial }{\partial y_{j}}\;g\left(f_{\left(h\right)}\right)\;=\;-2\;f_{j}\;u_{j},\\ d_{m+j}^{\left(\kappa \right)}\;:=\;\frac{\partial }{\partial y_{m+j}}\;g\left(f_{\left(h\right)}\right)\;=\;u_{j},\\ h_{j,k}^{\left(\kappa \right)}\;:=\;\frac{\partial^{2}}{\partial y_{j}\;\partial y_{k}}\;g\left(f_{\left(h\right)}\right)\;=2\;f_{j}\;f_{k}\;\left(\left(1-f_{k}\right)\;u_{j}\;v_{k}+\left(1-f_{j}\right)\;u_{k}\;v_{j}\right)\\ \;\;\;\;\;\;\;\;\;+\;2\;\left(f_{j}+f_{k}-4\;f_{j}\;f_{k}\right)\;u_{j}\;u_{k}\;-\;2\;\delta_{j,k}\;\left(u_{j}+2\;f_{j}\;v_{j}\right),\\ h_{j,m+k}^{\left(\kappa \right)}\;:=\;\frac{\partial^{2}}{\partial y_{j}\;\partial y_{m+k}}\;g\left(f_{\left(h\right)}\right)\;=\;\left(\delta_{j,k}-f_{j}\left(1-f_{j}\right)\;u_{k}\right)\;v_{j}\;-\;\left(1-2\;f_{j}\right)\;u_{j}\;u_{k},\\ h_{m+j,m+k}^{\left(\kappa \right)}\;:=\;\frac{\partial^{2}}{\partial y_{m+j}\;\partial y_{m+k}}\;g\left(f_{\left(h\right)}\right)\;=0. \end{array}$$

The proof of Equation (A2) is provided by Appendix A.2. We denote the gradient (Jacobian) of $g\left(f_{\left(h\right)}\right)$ by $D_{\kappa }=\left(d_{0}^{\left(\kappa \right)},\ldots ,d_{2\;m-1}^{\left(\kappa \right)}\right)$, and the Hessian equals $H_{\kappa }=diag\left(h_{00}^{\left(\kappa \right)},\ldots ,h_{2\;m-1,2\;m-1}^{\left(\kappa \right)}\right)$.

**Example** **A1.** *Let us pick up Example 1 and continue with the derivatives of the EGFs, as required for evaluating* (A1) *and* (A2).
*For $\phi_{a}\left(z\right)=\frac{z-z^{a}}{a-1}$, we have $\phi_{a}^{\prime \prime }\left(z\right)=-a\;z^{a-2}$, and thus $\phi_{a}^{\prime \prime \prime }\left(z\right)=-a\left(a-2\right)\;z^{a-3}$. Specific examples are*

- *a→1 corresponding to the EGF ϕ(z)=−zlnz, then*
  $$\phi^{\prime \prime }\left(z\right)=-\frac{1}{z},\;\phi^{\prime \prime }\left(z\right)=\frac{1}{z^{2}},\;\mathrm{so}\;u_{j}\;=\;\frac{1}{m}\;\frac{1}{f_{j}\left(1-f_{j}\right)},\;v_{j}\;=\;\frac{1}{m}\;\frac{2\;f_{j}-1}{f_{j}^{2}{\left(1-f_{j}\right)}^{2}};$$
- *a=2 corresponding to the EGF ϕ(z)=z(1−z), then*
  $$\phi^{\prime \prime }\left(z\right)=-2,\;\phi^{\prime \prime \prime }\left(z\right)=0,\;\mathrm{so}\;u_{j}\;=\;{\left(\sum_{i=0}^{m-1}f_{i}\left(1-f_{i}\right)\right)}^{-1},\;v_{j}\;=\;0;$$
- *a=3 corresponding to the EGF $\phi \left(z\right)=\frac{1}{2}\;z\left(1-z^{2}\right)$, then*
  $$\phi^{\prime \prime }\left(z\right)=-3\;z,\;\phi^{\prime \prime \prime }\left(z\right)=-3,\;\mathrm{so}\;u_{j},v_{j}\;\mathrm{are}\;\mathrm{as}\;\mathrm{before}.$$
  *For the equivalence of the a=2,3, recall (7).*

*For $\phi_{q}\left(z\right)=1-{\left(1-2\;\min\left\{z,1-z\right\}\right)}^{q}=1-{\left|2\;z-1\right|}^{q}$ with q>3, we get*

$$\begin{array}{cc} \phi_{q}^{\prime \prime }\left(z\right)= & {-4\;q\left(q-1\right)\;|2\;z-1|}^{q-2},\;\phi_{q}^{\prime \prime \prime }\left(z\right)=-8\;q\left(q-1\right)\left(q-2\right)\;{\left|2\;z-1\right|}^{q-3}\;\mathrm{sgn}\left(2\;z-1\right). \end{array}$$

*Recall that* (A1) *simplifies because of the symmetry of $\phi_{q}\left(z\right)$ around 1/2.*

Using (A2), the second-order Taylor expansion of ${\hat{\kappa }}_{\phi }\left(h\right)$ equals

(A3) $${\hat{\kappa }}_{\phi }\left(h\right)\;\approx \;\kappa_{\phi }\left(h\right)\;+\;D_{\kappa }\;\left({\hat{f}}_{\left(h\right)}-f_{\left(h\right)}\right)\;+\;\frac{1}{2}\;{\left({\hat{f}}_{\left(h\right)}-f_{\left(h\right)}\right)}^{⊤}\;H_{\kappa }\;\left({\hat{f}}_{\left(h\right)}-f_{\left(h\right)}\right).$$

In analogy to Section 3, we now conclude that

(A4) $$\begin{array}{c} \sqrt{n}\;({\hat{\kappa }}_{\phi }\left(h\right)-\kappa_{\phi }\left(h\right))\;\overset{d}{\to }\;N\left(0,\;\sigma_{\kappa }^{2}\right)\;\mathrm{with}\;\sigma_{\kappa }^{2}=D_{\kappa }\;\Sigma^{\left(h\right)}\;D_{\kappa }^{⊤},\\ \mathrm{where}\;\sigma_{\kappa }^{2}\;=\;\sum_{j,k=0}^{m-1}u_{j}\;u_{k}\;{\left(f_{\min\left\{j,k\right\}}-f_{j}\;f_{k}\right)}^{2}. \end{array}$$

The proof of Equation (A4) is provided by Appendix A.3. $\sigma_{\kappa }^{2}$ is computed explicitly by plugging-in (A1).

An approximate bias correction is obtained from (A3) (see the analogous arguments in Section 3). Using that $h_{m+j,m+k}^{\left(\kappa \right)}=0$, we get that

(A5) $$\begin{array}{c} E\left[{\hat{\kappa }}_{\phi }\left(h\right)\right]\;\approx \;0\;+\;\frac{1}{2\;n}\;\sum_{j,k=0}^{m-1}\left(h_{j,k}^{\left(\kappa \right)}\;\sigma_{j,k}\;+\;2\;h_{j,m+k}^{\left(\kappa \right)}\;\sigma_{j,m+k}^{\left(h\right)}\right)\;=\;-\frac{1}{n}, \end{array}$$

see the proof in Appendix A.4. Using (A4) and (A5), the derivation of Theorem 2 is complete.

### Appendix A.2. Proof of Equation (A2)

For

$$g\left(y\right)\;=\;\frac{\sum_{i=0}^{m-1}\left(\phi^{\prime \prime }\left(y_{i}\right)+\phi^{\prime \prime }\left(1-y_{i}\right)\right)\;\left(y_{m+i}-y_{i}^{2}\right)}{\sum_{i=0}^{m-1}\left(\phi^{\prime \prime }\left(y_{i}\right)+\phi^{\prime \prime }\left(1-y_{i}\right)\right)\;y_{i}\left(1-y_{i}\right)},$$

we compute all partial derivatives up to order 2. For doing this, we have to require that the EGF ϕ is even four times differentiable. Then,

$$\frac{\partial }{\partial y_{m+j}}\;g\left(y\right)\;=\;\frac{\phi^{\prime \prime }\left(y_{j}\right)+\phi^{\prime \prime }\left(1-y_{j}\right)}{\sum_{i=0}^{m-1}\left(\phi^{\prime \prime }\left(y_{i}\right)+\phi^{\prime \prime }\left(1-y_{i}\right)\right)\;y_{i}\left(1-y_{i}\right)}\;\mathrm{and}\;\frac{\partial^{2}}{\partial y_{m+j}\;\partial y_{m+k}}\;g\left(y\right)\;=\;0\;\mathrm{for}\;\mathrm{all}\;0\leq j,k\leq m-1.$$

Next, using the product and chain rule,

$$\frac{\partial }{\partial y_{j}}\;g\left(y\right)\;=\;\frac{\frac{\partial }{\partial y_{j}}\;\left(\phi^{\prime \prime }\left(y_{j}\right)+\phi^{\prime \prime }\left(1-y_{j}\right)\right)\;\left(y_{m+j}-y_{j}^{2}\right)}{\sum_{i=0}^{m-1}\left(\phi^{\prime \prime }\left(y_{i}\right)+\phi^{\prime \prime }\left(1-y_{i}\right)\right)\;y_{i}\left(1-y_{i}\right)}\;-\;\frac{g\left(y\right)\;\frac{\partial }{\partial y_{j}}\;\left(\phi^{\prime \prime }\left(y_{j}\right)+\phi^{\prime \prime }\left(1-y_{j}\right)\right)\;y_{j}\left(1-y_{j}\right)}{\sum_{i=0}^{m-1}\left(\phi^{\prime \prime }\left(y_{i}\right)+\phi^{\prime \prime }\left(1-y_{i}\right)\right)\;y_{i}\left(1-y_{i}\right)}.$$

Here,

$$\begin{array}{c} \frac{\partial }{\partial y_{j}}\;\left(\phi^{\prime \prime }\left(y_{j}\right)+\phi^{\prime \prime }\left(1-y_{j}\right)\right)\;\left(y_{m+j}-y_{j}^{2}\right)\\ \;=\;\left(\phi^{\prime \prime \prime }\left(y_{j}\right)-\phi^{\prime \prime \prime }\left(1-y_{j}\right)\right)\;\left(y_{m+j}-y_{j}^{2}\right)\;-\;2\;y_{j}\;\left(\phi^{\prime \prime }\left(y_{j}\right)+\phi^{\prime \prime }\left(1-y_{j}\right)\right), \end{array}$$

and

$$\begin{array}{c} \frac{\partial }{\partial y_{j}}\;\left(\phi^{\prime \prime }\left(y_{j}\right)+\phi^{\prime \prime }\left(1-y_{j}\right)\right)\;y_{j}\left(1-y_{j}\right)\\ \;=\;\left(\phi^{\prime \prime \prime }\left(y_{j}\right)-\phi^{\prime \prime \prime }\left(1-y_{j}\right)\right)\;y_{j}\left(1-y_{j}\right)\;+\;\left(1-2\;y_{j}\right)\;\left(\phi^{\prime \prime }\left(y_{j}\right)+\phi^{\prime \prime }\left(1-y_{j}\right)\right). \end{array}$$

Thus,

$$\begin{array}{cc} \frac{\partial }{\partial y_{j}}\;g\left(y\right)\;= & {\left(\sum_{i=0}^{m-1}\left(\phi^{\prime \prime }\left(y_{i}\right)+\phi^{\prime \prime }\left(1-y_{i}\right)\right)\;y_{i}\left(1-y_{i}\right)\right)}^{-1}\\ & \;\cdot \;((\phi^{\prime \prime \prime }\left(y_{j}\right)-\phi^{\prime \prime \prime }\left(1-y_{j}\right))\;(y_{m+j}-y_{j}^{2}\;-\;g\left(y\right)\;y_{j}\left(1-y_{j}\right))\\ & \;-\;(\phi^{\prime \prime }\left(y_{j}\right)+\phi^{\prime \prime }\left(1-y_{j}\right))\;(2\;y_{j}\;+\;g\left(y\right)\;\left(1-2\;y_{j}\right))). \end{array}$$

Consequently,

$$\begin{array}{c} \frac{\partial^{2}}{\partial y_{j}\;\partial y_{m+k}}\;g\left(y\right)\;=\;\frac{\delta_{j,k}\;\left(\phi^{\prime \prime \prime }\left(y_{j}\right)-\phi^{\prime \prime \prime }\left(1-y_{j}\right)\right)}{\sum_{i=0}^{m-1}\left(\phi^{\prime \prime }\left(y_{i}\right)+\phi^{\prime \prime }\left(1-y_{i}\right)\right)\;y_{i}\left(1-y_{i}\right)}\;-\;\frac{\frac{\partial }{\partial y_{m+k}}\;g\left(y\right)}{\sum_{i=0}^{m-1}\left(\phi^{\prime \prime }\left(y_{i}\right)+\phi^{\prime \prime }\left(1-y_{i}\right)\right)\;y_{i}\left(1-y_{i}\right)}\\ \;\cdot \;(\left(\phi^{\prime \prime \prime }\left(y_{j}\right)-\phi^{\prime \prime \prime }\left(1-y_{j}\right)\right)\;y_{j}\left(1-y_{j}\right)\;+\;\left(1-2\;y_{j}\right)\;\left(\phi^{\prime \prime }\left(y_{j}\right)+\phi^{\prime \prime }\left(1-y_{j}\right)\right)). \end{array}$$

Finally,

$$\begin{array}{c} \frac{\partial^{2}}{\partial y_{j}\;\partial y_{k}}\;g\left(y\right)\;=\;-\frac{\frac{\partial }{\partial y_{j}}\;g\left(y\right)\;\frac{\partial }{\partial y_{k}}\;\left(\phi^{\prime \prime }\left(y_{k}\right)+\phi^{\prime \prime }\left(1-y_{k}\right)\right)\;y_{k}\left(1-y_{k}\right)}{\sum_{i=0}^{m-1}\left(\phi^{\prime \prime }\left(y_{i}\right)+\phi^{\prime \prime }\left(1-y_{i}\right)\right)\;y_{i}\left(1-y_{i}\right)}\\ \;+\;{\left(\sum_{i=0}^{m-1}\left(\phi^{\prime \prime }\left(y_{i}\right)+\phi^{\prime \prime }\left(1-y_{i}\right)\right)\;y_{i}\left(1-y_{i}\right)\right)}^{-1}\\ \;\left.\begin{array}{c} \cdot \;\frac{\partial }{\partial y_{k}}\;(\left(\phi^{\prime \prime \prime }\left(y_{j}\right)-\phi^{\prime \prime \prime }\left(1-y_{j}\right)\right)\;\left(y_{m+j}-y_{j}^{2}\;-\;g\left(y\right)\;y_{j}\left(1-y_{j}\right)\right)\\ \;\;-\;\left(\phi^{\prime \prime }\left(y_{j}\right)+\phi^{\prime \prime }\left(1-y_{j}\right)\right)\;\left(2\;y_{j}\;+\;g\left(y\right)\;\left(1-2\;y_{j}\right)\right)) \end{array}\right\}=:A. \end{array}$$

Here, we have for k≠j that

$$\begin{array}{cc} A\;=\;-( & y_{j}\left(1-y_{j}\right)\;\left(\phi^{\prime \prime \prime }\left(y_{j}\right)-\phi^{\prime \prime \prime }\left(1-y_{j}\right)\right)\;+\;\left(1-2\;y_{j}\right)\;\left(\phi^{\prime \prime }\left(y_{j}\right)+\phi^{\prime \prime }\left(1-y_{j}\right)\right))\;\frac{\partial }{\partial y_{k}}\;g\left(y\right). \end{array}$$

For k=j, we have that

$$\begin{array}{cc} A\;= & \frac{\partial }{\partial y_{j}}\;\left(\phi^{\prime \prime \prime }\left(y_{j}\right)-\phi^{\prime \prime \prime }\left(1-y_{j}\right)\right)\;\left(y_{m+j}-y_{j}^{2}\;-\;g\left(y\right)\;y_{j}\left(1-y_{j}\right)\right)\\ & \;-\;\frac{\partial }{\partial y_{j}}\;\left(\phi^{\prime \prime }\left(y_{j}\right)+\phi^{\prime \prime }\left(1-y_{j}\right)\right)\;\left(2\;y_{j}\;+\;g\left(y\right)\;\left(1-2\;y_{j}\right)\right)\\ = & \left(\phi^{\left(4\right)}\left(y_{j}\right)+\phi^{\left(4\right)}\left(1-y_{j}\right)\right)\;\left(y_{m+j}-y_{j}^{2}\;-\;g\left(y\right)\;y_{j}\left(1-y_{j}\right)\right)\\ & \;-\;\left(\phi^{\prime \prime \prime }\left(y_{j}\right)-\phi^{\prime \prime \prime }\left(1-y_{j}\right)\right)\;y_{j}\left(1-y_{j}\right)\;\frac{\partial }{\partial y_{j}}\;g\left(y\right)\\ & \;-\;2\;\left(\phi^{\prime \prime \prime }\left(y_{j}\right)-\phi^{\prime \prime \prime }\left(1-y_{j}\right)\right)\;\left(2\;y_{j}\;+\;g\left(y\right)\;\left(1-2\;y_{j}\right)\right)\\ & \;-\;\left(\phi^{\prime \prime }\left(y_{j}\right)+\phi^{\prime \prime }\left(1-y_{j}\right)\right)\;\left(2-2\;g\left(y\right)\;+\;\left(1-2\;y_{j}\right)\;\frac{\partial }{\partial y_{j}}\;g\left(y\right)\right). \end{array}$$

So, altogether,

$$\begin{array}{cc} A\;= & -\delta_{jk}\;(2\;\left(\phi^{\prime \prime }\left(y_{j}\right)+\phi^{\prime \prime }\left(1-y_{j}\right)\right)\;\left(1-g(y)\right)\;+\;2\;\left(\phi^{\prime \prime \prime }\left(y_{j}\right)-\phi^{\prime \prime \prime }\left(1-y_{j}\right)\right)\;\left(2\;y_{j}\;+\;g\left(y\right)\;\left(1-2\;y_{j}\right)\right)\\ & \;\;-\;\left(\phi^{\left(4\right)}\left(y_{j}\right)+\phi^{\left(4\right)}\left(1-y_{j}\right)\right)\;\left(y_{m+j}-y_{j}^{2}\;-\;g\left(y\right)\;y_{j}\left(1-y_{j}\right)\right))\\ & -\;(y_{j}\left(1-y_{j}\right)\;\left(\phi^{\prime \prime \prime }\left(y_{j}\right)-\phi^{\prime \prime \prime }\left(1-y_{j}\right)\right)\;+\;\left(1-2\;y_{j}\right)\;\left(\phi^{\prime \prime }\left(y_{j}\right)+\phi^{\prime \prime }\left(1-y_{j}\right)\right))\;\frac{\partial }{\partial y_{k}}\;g\left(y\right). \end{array}$$

Hence,

$$\begin{array}{c} \frac{\partial^{2}}{\partial y_{j}\;\partial y_{k}}\;g\left(y\right)\;=\;-\frac{\left(y_{k}\left(1-y_{k}\right)\;\left(\phi^{\prime \prime \prime }\left(y_{k}\right)-\phi^{\prime \prime \prime }\left(1-y_{k}\right)\right)\;+\;\left(1-2\;y_{k}\right)\;\left(\phi^{\prime \prime }\left(y_{k}\right)+\phi^{\prime \prime }\left(1-y_{k}\right)\right)\right)\;\frac{\partial }{\partial y_{j}}\;g\left(y\right)}{\sum_{i=0}^{m-1}\left(\phi^{\prime \prime }\left(y_{i}\right)+\phi^{\prime \prime }\left(1-y_{i}\right)\right)\;y_{i}\left(1-y_{i}\right)}\\ \;-\;\frac{\left(y_{j}\left(1-y_{j}\right)\;\left(\phi^{\prime \prime \prime }\left(y_{j}\right)-\phi^{\prime \prime \prime }\left(1-y_{j}\right)\right)\;+\;\left(1-2\;y_{j}\right)\;\left(\phi^{\prime \prime }\left(y_{j}\right)+\phi^{\prime \prime }\left(1-y_{j}\right)\right)\right)\;\frac{\partial }{\partial y_{k}}\;g\left(y\right)}{\sum_{i=0}^{m-1}\left(\phi^{\prime \prime }\left(y_{i}\right)+\phi^{\prime \prime }\left(1-y_{i}\right)\right)\;y_{i}\left(1-y_{i}\right)}\\ \;-\;\delta_{jk}\;{\left(\sum_{i=0}^{m-1}\left(\phi^{\prime \prime }\left(y_{i}\right)+\phi^{\prime \prime }\left(1-y_{i}\right)\right)\;y_{i}\left(1-y_{i}\right)\right)}^{-1}\\ \;\cdot \;(2\;\left(\phi^{\prime \prime }\left(y_{j}\right)+\phi^{\prime \prime }\left(1-y_{j}\right)\right)\;\left(1-g(y)\right)\;+\;2\;\left(\phi^{\prime \prime \prime }\left(y_{j}\right)-\phi^{\prime \prime \prime }\left(1-y_{j}\right)\right)\;\left(2\;y_{j}\;+\;g\left(y\right)\;\left(1-2\;y_{j}\right)\right)\\ \;\;-\;\left(\phi^{\left(4\right)}\left(y_{j}\right)+\phi^{\left(4\right)}\left(1-y_{j}\right)\right)\;\left(y_{m+j}-y_{j}^{2}\;-\;g\left(y\right)\;y_{j}\left(1-y_{j}\right)\right)). \end{array}$$

For the required Taylor expansion, we have to evaluate all these derivatives in $y=f_{\left(h\right)}$, using that $f_{ii}\left(h\right)=f_{i}^{2}$ for all i=0,…,m−1, and thus $g\left(f_{\left(h\right)}\right)=0$. We use the notations introduced in (A1), i.e.,

$$u_{j}\;:=\;\frac{\phi^{\prime \prime }\left(f_{j}\right)+\phi^{\prime \prime }\left(1-f_{j}\right)}{\sum_{i=0}^{m-1}\left(\phi^{\prime \prime }\left(f_{i}\right)+\phi^{\prime \prime }\left(1-f_{i}\right)\right)\;f_{i}\left(1-f_{i}\right)},\;v_{j}\;:=\;\frac{\phi^{\prime \prime \prime }\left(f_{j}\right)-\phi^{\prime \prime \prime }\left(1-f_{j}\right)}{\sum_{i=0}^{m-1}\left(\phi^{\prime \prime }\left(f_{i}\right)+\phi^{\prime \prime }\left(1-f_{i}\right)\right)\;f_{i}\left(1-f_{i}\right)}.$$

Then, we get for 0≤j≤m−1 that

$$\begin{array}{cc} d_{j}^{\left(\kappa \right)}\;:= & \frac{\partial }{\partial y_{j}}\;g\left(f_{\left(h\right)}\right)\;=\;\frac{0\;-\;2\;f_{j}\;\left(\phi^{\prime \prime }\left(f_{j}\right)+\phi^{\prime \prime }\left(1-f_{j}\right)\right)}{\sum_{i=0}^{m-1}\left(\phi^{\prime \prime }\left(f_{i}\right)+\phi^{\prime \prime }\left(1-f_{i}\right)\right)\;f_{i}\left(1-f_{i}\right)}\;=\;-2\;f_{j}\;u_{j},\\ d_{m+j}^{\left(\kappa \right)}\;:= & \frac{\partial }{\partial y_{m+j}}\;g\left(f_{\left(h\right)}\right)\;=\;u_{j}. \end{array}$$

For the second-order derivatives, we get for 0≤j,k≤m−1 that

$$\begin{array}{cc} h_{j,k}^{\left(\kappa \right)}\;:= & \frac{\partial^{2}}{\partial y_{j}\;\partial y_{k}}\;g\left(f_{\left(h\right)}\right)\\ = & -\frac{\left(f_{k}\left(1-f_{k}\right)\;\left(\phi^{\prime \prime \prime }\left(f_{k}\right)-\phi^{\prime \prime \prime }\left(1-f_{k}\right)\right)\;+\;\left(1-2\;f_{k}\right)\;\left(\phi^{\prime \prime }\left(f_{k}\right)+\phi^{\prime \prime }\left(1-f_{k}\right)\right)\right)\;d_{j}^{\left(\kappa \right)}}{\sum_{i=0}^{m-1}\left(\phi^{\prime \prime }\left(f_{i}\right)+\phi^{\prime \prime }\left(1-f_{i}\right)\right)\;f_{i}\left(1-f_{i}\right)}\\ & \;-\;\frac{\left(f_{j}\left(1-f_{j}\right)\;\left(\phi^{\prime \prime \prime }\left(f_{j}\right)-\phi^{\prime \prime \prime }\left(1-f_{j}\right)\right)\;+\;\left(1-2\;f_{j}\right)\;\left(\phi^{\prime \prime }\left(f_{j}\right)+\phi^{\prime \prime }\left(1-f_{j}\right)\right)\right)\;d_{k}^{\left(\kappa \right)}}{\sum_{i=0}^{m-1}\left(\phi^{\prime \prime }\left(f_{i}\right)+\phi^{\prime \prime }\left(1-f_{i}\right)\right)\;f_{i}\left(1-f_{i}\right)}\\ & \;-\;\frac{\delta_{jk}\;2\;\left(\left(\phi^{\prime \prime }\left(f_{j}\right)+\phi^{\prime \prime }\left(1-f_{j}\right)\right)\;+\;2\;f_{j}\;\left(\phi^{\prime \prime \prime }\left(f_{j}\right)-\phi^{\prime \prime \prime }\left(1-f_{j}\right)\right)\right)}{\sum_{i=0}^{m-1}\left(\phi^{\prime \prime }\left(f_{i}\right)+\phi^{\prime \prime }\left(1-f_{i}\right)\right)\;f_{i}\left(1-f_{i}\right)}\\ = & -f_{k}\left(1-f_{k}\right)\;v_{k}\;d_{j}^{\left(\kappa \right)}\;-\;\left(1-2\;f_{k}\right)\;u_{k}\;d_{j}^{\left(\kappa \right)}\\ & \;-\;f_{j}\left(1-f_{j}\right)\;v_{j}\;d_{k}^{\left(\kappa \right)}\;-\;\left(1-2\;f_{j}\right)\;u_{j}\;d_{k}^{\left(\kappa \right)}\;-\;2\;\delta_{jk}\;u_{j}\;-\;4\;\delta_{jk}\;f_{j}\;v_{j}\\ = & -f_{k}\left(1-f_{k}\right)\;\left(-2\;f_{j}\;u_{j}\right)\;v_{k}\;-\;\left(1-2\;f_{k}\right)\;\left(-2\;f_{j}\;u_{j}\right)\;u_{k}\\ & \;-\;f_{j}\left(1-f_{j}\right)\;\left(-2\;f_{k}\;u_{k}\right)\;v_{j}\;-\;\left(1-2\;f_{j}\right)\;\left(-2\;f_{k}\;u_{k}\right)\;u_{j}\;-\;2\;\delta_{jk}\;\left(u_{j}+2\;f_{j}\;v_{j}\right)\\ = & 2\;f_{j}\;f_{k}\;\left(1-f_{k}\right)\;u_{j}\;v_{k}\;+\;2\;f_{j}\;f_{k}\;\left(1-f_{j}\right)\;u_{k}\;v_{j}\\ & \;+\;2\;f_{j}\;\left(1-2\;f_{k}\right)\;u_{j}\;u_{k}\;+\;2\;f_{k}\;\left(1-2\;f_{j}\right)\;u_{j}\;u_{k}\;-\;2\;\delta_{jk}\;\left(u_{j}+2\;f_{j}\;v_{j}\right)\\ = & 2\;f_{j}\;f_{k}\;\left(\left(1-f_{k}\right)\;u_{j}\;v_{k}+\left(1-f_{j}\right)\;u_{k}\;v_{j}\right)\;+\;2\;\left(f_{j}+f_{k}-4\;f_{j}\;f_{k}\right)\;u_{j}\;u_{k}\;-\;2\;\delta_{j,k}\;\left(u_{j}+2\;f_{j}\;v_{j}\right). \end{array}$$

Similarly,

$$\begin{array}{cc} h_{j,m+k}^{\left(\kappa \right)}\;:= & \frac{\partial^{2}}{\partial y_{j}\;\partial y_{m+k}}\;g\left(f_{\left(h\right)}\right)\;=\;\frac{\delta_{j,k}\;\left(\phi^{\prime \prime \prime }\left(f_{j}\right)-\phi^{\prime \prime \prime }\left(1-f_{j}\right)\right)}{\sum_{i=0}^{m-1}\left(\phi^{\prime \prime }\left(f_{i}\right)+\phi^{\prime \prime }\left(1-f_{i}\right)\right)\;f_{i}\left(1-f_{i}\right)}\\ & \;-\;\frac{d_{m+k}^{\left(\kappa \right)}\;\left(\phi^{\prime \prime \prime }\left(f_{j}\right)-\phi^{\prime \prime \prime }\left(1-f_{j}\right)\right)\;f_{j}\left(1-f_{j}\right)}{\sum_{i=0}^{m-1}\left(\phi^{\prime \prime }\left(f_{i}\right)+\phi^{\prime \prime }\left(1-f_{i}\right)\right)\;f_{i}\left(1-f_{i}\right)}\;-\;\frac{d_{m+k}^{\left(\kappa \right)}\;\left(1-2\;f_{j}\right)\;\left(\phi^{\prime \prime }\left(f_{j}\right)+\phi^{\prime \prime }\left(1-f_{j}\right)\right)}{\sum_{i=0}^{m-1}\left(\phi^{\prime \prime }\left(f_{i}\right)+\phi^{\prime \prime }\left(1-f_{i}\right)\right)\;f_{i}\left(1-f_{i}\right)}\\ = & \left(\delta_{j,k}-d_{m+k}^{\left(\kappa \right)}\;f_{j}\left(1-f_{j}\right)\right)\;v_{j}\;-\;d_{m+k}^{\left(\kappa \right)}\;\left(1-2\;f_{j}\right)\;u_{j}\\ = & \left(\delta_{j,k}-f_{j}\left(1-f_{j}\right)\;u_{k}\right)\;v_{j}\;-\;\left(1-2\;f_{j}\right)\;u_{j}\;u_{k}. \end{array}$$

Finally,

$$h_{m+j,m+k}^{\left(\kappa \right)}\;:=\;\frac{\partial^{2}}{\partial y_{m+j}\;\partial y_{m+k}}\;g\left(f_{\left(h\right)}\right)\;=0.$$

This completes the proof of (A2).

### Appendix A.3. Proof of Equation (A4)

$\sigma_{\kappa }^{2}=D_{\kappa }\;\Sigma^{\left(h\right)}\;D_{\kappa }^{⊤}$ is computed as follows:

$$\begin{array}{cc} \sigma_{\kappa }^{2}\;= & \sum_{j,k=0}^{m-1}\left(d_{j}^{\left(\kappa \right)}\;d_{k}^{\left(\kappa \right)}\;\sigma_{j,k}\;+\;2\;d_{j}^{\left(\kappa \right)}\;d_{m+k}^{\left(\kappa \right)}\;\sigma_{j,m+k}^{\left(h\right)}\;+\;d_{m+j}^{\left(\kappa \right)}\;d_{m+k}^{\left(\kappa \right)}\;\sigma_{m+j,m+k}^{\left(h\right)}\right)\\ \;\overset{\left(A2\right)}{=} & \sum_{j,k=0}^{m-1}\left(4\;f_{j}\;f_{k}\;u_{j}\;u_{k}\;\sigma_{j,k}\;-\;4\;f_{j}\;u_{j}\;u_{k}\;\sigma_{j,m+k}^{\left(h\right)}\;+\;u_{j}\;u_{k}\;\sigma_{m+j,m+k}^{\left(h\right)}\right)\\ \;\overset{\left(17\right)}{=} & \sum_{j,k=0}^{m-1}(-4\;f_{j}\;f_{k}\;u_{j}\;u_{k}\;\left(f_{\min\left\{j,k\right\}}-f_{j}\;f_{k}\right)\;+\;u_{j}\;u_{k}\;\left(f_{\min\left\{j,k\right\}}+3\;f_{j}\;f_{k}\right)\;\left(f_{\min\left\{j,k\right\}}-f_{j}\;f_{k}\right))\\ \;= & \sum_{j,k=0}^{m-1}u_{j}\;u_{k}\;{\left(f_{\min\left\{j,k\right\}}-f_{j}\;f_{k}\right)}^{2}. \end{array}$$

This completes the proof of (A4).

### Appendix A.4. Proof of Equation (A5)

Using that $h_{m+j,m+k}^{\left(\kappa \right)}=0$, we get that

$$\begin{array}{cc} E\left[{\hat{\kappa }}_{\phi }\left(h\right)\right]\;\approx & 0\;+\;\frac{1}{2\;n}\;\sum_{j,k=0}^{m-1}\left(h_{j,k}^{\left(\kappa \right)}\;\sigma_{j,k}\;+\;2\;h_{j,m+k}^{\left(\kappa \right)}\;\sigma_{j,m+k}^{\left(h\right)}\right)\\ \;\overset{\left(17\right)}{=} & \frac{1}{2\;n}\;\sum_{j,k=0}^{m-1}\sigma_{j,k}\;\left(h_{j,k}^{\left(\kappa \right)}\;+\;4\;f_{k}\;h_{j,m+k}^{\left(\kappa \right)}\right)\\ \;\overset{\left(A2\right)}{=} & \frac{1}{2\;n}\;\sum_{j,k=0}^{m-1}\sigma_{j,k}\;(2\;f_{j}\;f_{k}\;\left(\left(1-f_{k}\right)\;u_{j}\;v_{k}+\left(1-f_{j}\right)\;u_{k}\;v_{j}\right)\\ & \;+\;2\;\left(f_{j}+f_{k}-4\;f_{j}\;f_{k}\right)\;u_{j}\;u_{k}\;-\;2\;\delta_{j,k}\;\left(u_{j}+2\;f_{j}\;v_{j}\right)\\ & \;+\;4\;f_{k}\;\left(\delta_{j,k}-f_{j}\left(1-f_{j}\right)\;u_{k}\right)\;v_{j}\;-\;4\;f_{k}\;\left(1-2\;f_{j}\right)\;u_{j}\;u_{k})\\ \;= & \frac{1}{2\;n}\;\sum_{j,k=0}^{m-1}\sigma_{j,k}\;(4\;f_{j}\;f_{k}\;\left(1-f_{j}\right)\;u_{k}\;v_{j}\;+\;4\;f_{k}\;\left(1-2\;f_{j}\right)\;u_{j}\;u_{k}\;-\;2\;\delta_{j,k}\;u_{j}\;-\;4\;\delta_{j,k}\;f_{j}\;v_{j}\\ & \;+\;4\;\delta_{j,k}\;f_{j}\;v_{j}\;-\;4\;f_{k}\;f_{j}\left(1-f_{j}\right)\;u_{k}\;v_{j}\;-\;4\;f_{k}\;\left(1-2\;f_{j}\right)\;u_{j}\;u_{k})\\ \;= & \frac{1}{2\;n}\;\sum_{j,k=0}^{m-1}\sigma_{j,k}\;\left(0\;-\;2\;\delta_{j,k}\;u_{j}\right)\;=\;-\frac{1}{n}\;\sum_{j=0}^{m-1}u_{j}\;\sigma_{j,j}\;=\;-\frac{1}{n}, \end{array}$$

where the last equality follows from (A1) by using $\sigma_{j,j}=f_{j}\left(1-f_{j}\right)$ according to (17). This completes the proof of (A5).

## Appendix B. Tables

**Table A1:** Simulated vs. asymptotic mean and SE of ${\hat{\mathrm{CPE}}}_{\phi }$ for specific cases of $\phi_{a}\left(z\right)=\left(z-z^{a}\right)/\left(a-1\right)$ and $\phi_{q}\left(z\right)=1-{\left|2\;z-1\right|}^{q}$, where i. i. d. rank counts $I_{t}∼\mathrm{Bin}\left(4,p\right)$ and sample size *n*.

		Simulated Mean					Asymptotic Mean				
p	n	a=1	a=1.5	a=2	a=2.5	q=4	a=1	a=1.5	a=2	a=2.5	q=4
0.1	50	0.305	0.282	0.273	0.272	0.336	0.301	0.282	0.273	0.272	0.337
	100	0.310	0.285	0.276	0.274	0.341	0.308	0.285	0.276	0.274	0.341
	250	0.313	0.287	0.278	0.276	0.343	0.312	0.287	0.278	0.276	0.343
	500	0.314	0.288	0.278	0.277	0.344	0.314	0.288	0.278	0.277	0.344
	1000	0.314	0.288	0.279	0.277	0.344	0.315	0.288	0.279	0.277	0.344
0.3	50	0.538	0.500	0.484	0.481	0.610	0.538	0.500	0.484	0.481	0.610
	100	0.546	0.506	0.490	0.486	0.618	0.545	0.505	0.489	0.486	0.617
	250	0.550	0.508	0.492	0.488	0.622	0.550	0.509	0.492	0.489	0.622
	500	0.551	0.509	0.493	0.489	0.624	0.551	0.510	0.493	0.490	0.624
	1000	0.552	0.510	0.494	0.490	0.625	0.552	0.510	0.494	0.490	0.625
0.5	50	0.601	0.554	0.536	0.532	0.679	0.602	0.555	0.536	0.532	0.679
	100	0.609	0.560	0.541	0.537	0.687	0.609	0.561	0.541	0.537	0.688
	250	0.613	0.564	0.544	0.540	0.693	0.614	0.564	0.545	0.540	0.693
	500	0.616	0.566	0.546	0.542	0.696	0.615	0.565	0.546	0.542	0.695
	1000	0.616	0.566	0.546	0.542	0.696	0.616	0.566	0.546	0.542	0.696
0.1	50	0.048	0.044	0.043	0.043	0.053	0.049	0.044	0.043	0.043	0.054
	100	0.034	0.031	0.030	0.030	0.037	0.034	0.031	0.030	0.030	0.038
	250	0.022	0.020	0.019	0.019	0.024	0.022	0.020	0.019	0.019	0.024
	500	0.015	0.014	0.014	0.014	0.017	0.015	0.014	0.014	0.014	0.017
	1000	0.011	0.010	0.010	0.009	0.012	0.011	0.010	0.010	0.010	0.012
0.3	50	0.053	0.051	0.051	0.050	0.059	0.053	0.051	0.051	0.051	0.058
	100	0.037	0.036	0.036	0.036	0.041	0.037	0.036	0.036	0.036	0.041
	250	0.024	0.023	0.023	0.023	0.026	0.024	0.023	0.023	0.023	0.026
	500	0.017	0.016	0.016	0.016	0.018	0.017	0.016	0.016	0.016	0.018
	1000	0.012	0.011	0.011	0.011	0.013	0.012	0.011	0.011	0.011	0.013
0.5	50	0.056	0.055	0.054	0.054	0.065	0.055	0.055	0.054	0.054	0.065
	100	0.039	0.038	0.038	0.038	0.046	0.039	0.039	0.038	0.038	0.046
	250	0.025	0.025	0.025	0.025	0.029	0.024	0.025	0.024	0.024	0.029
	500	0.017	0.017	0.017	0.017	0.021	0.017	0.017	0.017	0.017	0.021
	1000	0.012	0.012	0.012	0.012	0.015	0.012	0.012	0.012	0.012	0.015

**Table A2:** Simulated vs. asymptotic mean and SE of ${\hat{\mathrm{CPE}}}_{\phi }$ for specific cases of $\phi_{a}\left(z\right)=\left(z-z^{a}\right)/\left(a-1\right)$ and $\phi_{q}\left(z\right)=1-{\left|2\;z-1\right|}^{q}$, where BAR(1) rank counts $I_{t}∼\mathrm{Bin}\left(4,p\right)$ with ρ=0.4 and sample size *n*.

		Simulated Mean					Asymptotic Mean				
p	n	a=1	a=1.5	a=2	a=2.5	q=4	a=1	a=1.5	a=2	a=2.5	q=4
0.1	50	0.298	0.276	0.268	0.266	0.330	0.292	0.275	0.267	0.266	0.330
	100	0.306	0.282	0.273	0.271	0.337	0.304	0.282	0.273	0.271	0.337
	250	0.312	0.286	0.277	0.275	0.342	0.311	0.286	0.277	0.275	0.342
	500	0.313	0.287	0.278	0.276	0.343	0.313	0.287	0.278	0.276	0.343
	1000	0.314	0.288	0.279	0.277	0.344	0.314	0.288	0.278	0.277	0.344
0.3	50	0.529	0.491	0.476	0.473	0.598	0.528	0.491	0.476	0.472	0.597
	100	0.540	0.501	0.485	0.481	0.611	0.540	0.501	0.485	0.481	0.611
	250	0.547	0.506	0.490	0.487	0.620	0.548	0.507	0.490	0.487	0.620
	500	0.551	0.509	0.493	0.489	0.623	0.550	0.509	0.492	0.489	0.623
	1000	0.552	0.510	0.493	0.490	0.624	0.551	0.510	0.493	0.490	0.624
0.5	50	0.590	0.545	0.527	0.523	0.667	0.592	0.545	0.527	0.523	0.666
	100	0.604	0.555	0.537	0.532	0.682	0.604	0.556	0.537	0.533	0.682
	250	0.612	0.562	0.543	0.539	0.691	0.612	0.562	0.543	0.539	0.691
	500	0.614	0.564	0.545	0.540	0.694	0.614	0.564	0.545	0.541	0.694
	1000	0.615	0.565	0.546	0.542	0.695	0.615	0.566	0.546	0.542	0.695
0.1	50	0.066	0.062	0.061	0.061	0.070	0.070	0.065	0.064	0.064	0.073
	100	0.047	0.045	0.044	0.044	0.050	0.049	0.046	0.045	0.045	0.052
	250	0.031	0.029	0.028	0.028	0.032	0.031	0.029	0.029	0.029	0.033
	500	0.022	0.020	0.020	0.020	0.023	0.022	0.021	0.020	0.020	0.023
	1000	0.015	0.014	0.014	0.014	0.016	0.016	0.015	0.014	0.014	0.016
0.3	50	0.064	0.061	0.061	0.060	0.071	0.065	0.063	0.062	0.062	0.073
	100	0.046	0.044	0.043	0.043	0.050	0.046	0.044	0.044	0.044	0.051
	250	0.029	0.028	0.027	0.027	0.032	0.029	0.028	0.028	0.028	0.032
	500	0.021	0.020	0.019	0.019	0.023	0.021	0.020	0.020	0.020	0.023
	1000	0.014	0.014	0.014	0.014	0.016	0.015	0.014	0.014	0.014	0.016
0.5	50	0.065	0.063	0.062	0.062	0.074	0.064	0.064	0.064	0.064	0.076
	100	0.046	0.046	0.045	0.045	0.054	0.045	0.046	0.045	0.045	0.054
	250	0.029	0.029	0.029	0.029	0.034	0.029	0.029	0.029	0.028	0.034
	500	0.020	0.020	0.020	0.020	0.024	0.020	0.020	0.020	0.020	0.024
	1000	0.014	0.014	0.014	0.014	0.017	0.014	0.014	0.014	0.014	0.017

**Table A3:** Simulated vs. asymptotic mean and SE of ${\hat{\kappa }}_{\phi }\left(1\right)$ for specific cases of $\phi_{a}\left(z\right)=\left(z-z^{a}\right)/\left(a-1\right)$ and $\phi_{q}\left(z\right)=1-{\left|2\;z-1\right|}^{q}$, where i. i. d. rank counts $I_{t}∼\mathrm{Bin}\left(4,p\right)$ and sample size *n*.

		Simulated Mean					Asymptotic Mean				
p	n	a=1	a=1.5	a=2	a=2.5	q=4	a=1	a=1.5	a=2	a=2.5	q=4
0.1	50	−0.020	−0.019	−0.019	−0.019	−0.020	−0.020	−0.020	−0.020	−0.020	−0.020
	100	0.005	−0.008	−0.008	−0.008	−0.009	−0.010	−0.010	−0.010	−0.010	−0.010
	250	0.003	−0.004	−0.004	−0.004	−0.004	−0.004	−0.004	−0.004	−0.004	−0.004
	500	0.000	−0.001	−0.002	−0.002	−0.001	−0.002	−0.002	−0.002	−0.002	−0.002
	1000	−0.001	−0.001	−0.001	−0.001	−0.001	−0.001	−0.001	−0.001	−0.001	−0.001
0.3	50	−0.020	−0.020	−0.020	−0.020	−0.020	−0.020	−0.020	−0.020	−0.020	−0.020
	100	−0.010	−0.010	−0.010	−0.010	−0.011	−0.010	−0.010	−0.010	−0.010	−0.010
	250	−0.003	−0.004	−0.004	−0.004	−0.003	−0.004	−0.004	−0.004	−0.004	−0.004
	500	−0.002	−0.002	−0.002	−0.002	−0.002	−0.002	−0.002	−0.002	−0.002	−0.002
	1000	−0.001	−0.001	−0.001	−0.001	−0.001	−0.001	−0.001	−0.001	−0.001	−0.001
0.5	50	−0.020	−0.021	−0.021	−0.021	−0.020	−0.020	−0.020	−0.020	−0.020	−0.020
	100	−0.010	−0.010	−0.010	−0.010	−0.010	−0.010	−0.010	−0.010	−0.010	−0.010
	250	−0.004	−0.004	−0.004	−0.004	−0.004	−0.004	−0.004	−0.004	−0.004	−0.004
	500	−0.002	−0.002	−0.002	−0.002	−0.002	−0.002	−0.002	−0.002	−0.002	−0.002
	1000	−0.001	−0.001	−0.001	−0.001	−0.001	−0.001	−0.001	−0.001	−0.001	−0.001
0.1	50	0.124	0.124	0.130	0.132	0.134	0.074	0.105	0.120	0.122	0.103
	100	0.134	0.082	0.088	0.089	0.085	0.053	0.074	0.085	0.086	0.073
	250	0.086	0.050	0.055	0.056	0.050	0.033	0.047	0.054	0.055	0.046
	500	0.045	0.034	0.038	0.039	0.034	0.023	0.033	0.038	0.039	0.033
	1000	0.022	0.024	0.027	0.027	0.023	0.017	0.023	0.027	0.027	0.023
0.3	50	0.098	0.097	0.101	0.101	0.101	0.079	0.089	0.096	0.097	0.088
	100	0.066	0.067	0.070	0.071	0.067	0.056	0.063	0.068	0.068	0.062
	250	0.040	0.040	0.043	0.043	0.040	0.035	0.040	0.043	0.043	0.040
	500	0.026	0.028	0.030	0.031	0.028	0.025	0.028	0.030	0.031	0.028
	1000	0.018	0.020	0.021	0.021	0.020	0.018	0.020	0.021	0.022	0.020
0.5	50	0.084	0.092	0.098	0.099	0.089	0.080	0.086	0.092	0.094	0.080
	100	0.058	0.063	0.067	0.068	0.059	0.057	0.061	0.065	0.066	0.057
	250	0.036	0.039	0.042	0.043	0.037	0.036	0.039	0.041	0.042	0.036
	500	0.025	0.027	0.029	0.030	0.025	0.025	0.027	0.029	0.030	0.025
	1000	0.018	0.019	0.021	0.021	0.018	0.018	0.019	0.021	0.021	0.018

**Table A4:** Simulated rejection rate (ρ=0: size; ρ≠0: power) of ${\hat{\kappa }}_{\phi }\left(1\right)$-test at 5 %-level ($H_{0}$: ρ=0, i.e., i. i. d.-case) for specific cases of $\phi_{a}\left(z\right)=\left(z-z^{a}\right)/\left(a-1\right)$ and $\phi_{q}\left(z\right)=1-{\left|2\;z-1\right|}^{q}$, where BAR(1) rank counts $I_{t}∼\mathrm{Bin}\left(4,0.3\right)$ and sample size *n*.

	Simulated Rejection Rate						Simulated Rejection Rate				
p	n	a=1	a=1.5	a=2	a=2.5	q=4	a=1	a=1.5	a=2	a=2.5	q=4
							ρ=0.2				
						50	0.227	0.265	0.263	0.262	0.222
						100	0.367	0.447	0.451	0.450	0.370
						250	0.721	0.830	0.830	0.829	0.723
						500	0.950	0.982	0.982	0.981	0.947
						1000	1.000	1.000	1.000	1.000	0.999
	ρ=−0.4						ρ=0.4				
50	0.484	0.565	0.585	0.588	0.312	50	0.663	0.749	0.756	0.757	0.625
100	0.807	0.878	0.891	0.893	0.672	100	0.917	0.959	0.962	0.962	0.897
250	0.989	0.999	1.000	1.000	0.987	250	1.000	1.000	1.000	1.000	0.999
500	0.997	1.000	1.000	1.000	1.000	500	1.000	1.000	1.000	1.000	1.000
1000	1.000	1.000	1.000	1.000	1.000	1000	1.000	1.000	1.000	1.000	1.000
	ρ=−0.2						ρ=0.6				
50	0.151	0.188	0.195	0.196	0.094	50	0.956	0.979	0.981	0.981	0.923
100	0.298	0.365	0.374	0.375	0.231	100	1.000	1.000	1.000	1.000	0.998
250	0.643	0.744	0.751	0.751	0.584	250	1.000	1.000	1.000	1.000	1.000
500	0.925	0.965	0.966	0.965	0.900	500	1.000	1.000	1.000	1.000	1.000
1000	0.994	1.000	1.000	1.000	0.998	1000	1.000	1.000	1.000	1.000	1.000
	ρ=0						ρ=0.8				
50	*0.061*	*0.056*	*0.054*	*0.054*	*0.061*	50	0.997	1.000	1.000	1.000	0.981
100	*0.056*	*0.060*	*0.058*	*0.057*	*0.057*	100	1.000	1.000	1.000	1.000	0.999
250	*0.047*	*0.048*	*0.046*	*0.046*	*0.049*	250	1.000	1.000	1.000	1.000	1.000
500	*0.049*	*0.050*	*0.050*	*0.050*	*0.052*	500	1.000	1.000	1.000	1.000	1.000
1000	*0.053*	*0.047*	*0.048*	*0.049*	*0.050*	1000	1.000	1.000	1.000	1.000	1.000
